# The microcephaly gene *Donson* is essential for progenitors of cortical glutamatergic and GABAergic neurons

**DOI:** 10.1371/journal.pgen.1009441

**Published:** 2021-03-19

**Authors:** Sathish Venkataramanappa, Dagmar Schütz, Friederike Saaber, Praveen Ashok Kumar, Philipp Abe, Stefan Schulz, Ralf Stumm

**Affiliations:** Institute of Pharmacology and Toxicology, Jena University Hospital, Jena, Germany; Duke-NUS Medical School, SINGAPORE

## Abstract

Biallelic mutations in *DONSON*, an essential gene encoding for a replication fork protection factor, were linked to skeletal abnormalities and microcephaly. To better understand DONSON function in corticogenesis, we characterized *Donson* expression and consequences of conditional *Donson* deletion in the mouse telencephalon. *Donson* was widely expressed in the proliferation and differentiation zones of the embryonic dorsal and ventral telencephalon, which was followed by a postnatal expression decrease. *Emx1*-Cre-mediated *Donson* deletion in progenitors of cortical glutamatergic neurons caused extensive apoptosis in the early dorsomedial neuroepithelium, thus preventing formation of the neocortex and hippocampus. At the place of the missing lateral neocortex, these mutants exhibited a dorsal extension of an early-generated paleocortex. Targeting cortical neurons at the intermediate progenitor stage using *Tbr2*-Cre evoked no apparent malformations, whereas *Nkx2*.*1*-Cre-mediated *Donson* deletion in subpallial progenitors ablated 75% of *Nkx2*.*1*-derived cortical GABAergic neurons. Thus, the early telencephalic neuroepithelium depends critically on Donson function. Our findings help explain why the neocortex is most severely affected in individuals with *DONSON* mutations and suggest that DONSON-dependent microcephaly might be associated with so far unrecognized defects in cortical GABAergic neurons. Targeting *Donson* using an appropriate recombinase is proposed as a feasible strategy to ablate proliferating and nascent cells in experimental research.

## Author summary

The cerebral cortex constitutes the largest part of the mammalian brain and is generated prenatally by highly proliferative progenitors. Genes encoding proteins that are essential for chromosomal segregation, mitotic division, DNA repair, and DNA damage response are frequently mutated in individuals diagnosed with microcephaly, a clinical condition characterized by cerebrocortical hypotrophy. Recent findings suggest that biallelic mutations in *DONSON*, a replication fork stabilization factor, cause microcephaly and skeletal defects, but this has not been formally tested. Here, we find that Cre-mediated *Donson* deletion in progenitors of cortical glutamatergic and cortical GABAergic neurons causes extensive programmed cell death at early stages of cortical development in mice. Cell death is induced in the proliferation zones and the postmitotic differentiation zones of the targeted progenitors. Mice undergoing *Donson* ablation in glutamatergic progenitors do not develop the hippocampus and dorsolateral neocortex, which leads to a dorsal shift of the early-generated piriform cortex. *Donson* deletion in GABAergic progenitors eliminates the vast majority of GABAergic neurons and oligodendrocyte precursors arising in the targeted lineage. We thus establish that *Donson* is essential for diverse early telencephalic progenitors. Targeting *Donson* might be used to kill off highly proliferating cells in experimental and probably therapeutic settings.

## Introduction

Primary microcephaly (PM) is a clinical neurodevelopmental condition defined by a congenital reduction in head circumference of at least 2 standard deviations below the ethnically matched, age- and sex-related mean. PM is usually transmitted as autosomal recessive trait with an incidence from 1.3 to 150 per 100,000 depending on the type of population and definition of PM. Clinical symptoms include a sloping forehead, reduced volume of the brain—especially of the cerebral cortex, non-progressive mental retardation, movement and feeding disorders, as well as short stature and epilepsy in some cases [1,2]. Up to date, 20 genes were linked to PM, most of which enable cells to execute precise chromosomal segregation and mitotic division by regulating mitotic spindle assembly, centriole duplication, mitotic checkpoint activity, DNA repair, and DNA damage response [2,3].

Genetic defects leading to PM are thought to affect neurogenesis by decreasing the pool of neural progenitors [4]. Since the cerebral cortex constitutes the largest part of the mammalian brain volume and neurogenesis is largely completed before birth, cortical progenitors need to generate neurons with high efficiency, which may render them particularly susceptible to defective cell cycle regulation [3]. Progenitors in the telencephalon consist of different types, namely neuroepithelial cells, radial-glial cells (RGC), and intermediate progenitor cells (IPC). Cortical size depends on the expansion of the progenitor pool by symmetric division, which prevails at early developmental stages and is carried out by the former two progenitor types [5]. IPC arise from neuroepithelial cells and RGC by asymmetric division and function as transit amplifying cells that increase neuronal output [6–11]. Once the cortical network is fully developed, it consists roughly of 80% glutamatergic excitatory neurons (cEN) and 20% GABAergic inhibitory neurons (cIN). The latter population originates in the medial and caudal ganglionic eminence (mGE, cGE) in the subpallium and needs to migrate extensively to reach all cortical areas [12]. Since the correct ratio of cEN versus cIN is essential for proper cortical functioning [13], it is of interest whether a gene implicated in PM is equally important for progenitors of cEN and progenitors of cIN.

DONSON is a replisome component protecting the replication fork and telomere ends [14,15]. In a seminal report, biallelic *DONSON* mutations were identified in 29 individuals with microcephalic primordial dwarfism [14]. The developmental defects were attributed to decreased checkpoint activity and chromosomal instability due to impaired DONSON function. Subsequent studies linked *DONSON* mutations to micromelia syndrome, Meier-Gorlin syndrome, Seckel-like syndrome, Femoral Facial syndrome, and microcephaly, short stature and limb abnormalities, which are all characterized by microcephaly as well as skeletal and craniofacial abnormalities [16–21]. The wealth of clinical data indicating that *DONSON* mutations can lead to severe developmental defects are opposed by the lack of studies assessing *DONSON* function by means of targeted gene deletion in model organisms. Since genome-wide deletion of *Donson* is lethal early in embryonic development [16], we employed conditional approaches. We find that both the early neuroepithelium generating the neocortex and hippocampus and progenitors generating cIN are highly sensitive to loss of *Donson* function.

## Results

### *Donson* is expressed in the embryonic and early postnatal telencephalon

**Fig 1:**
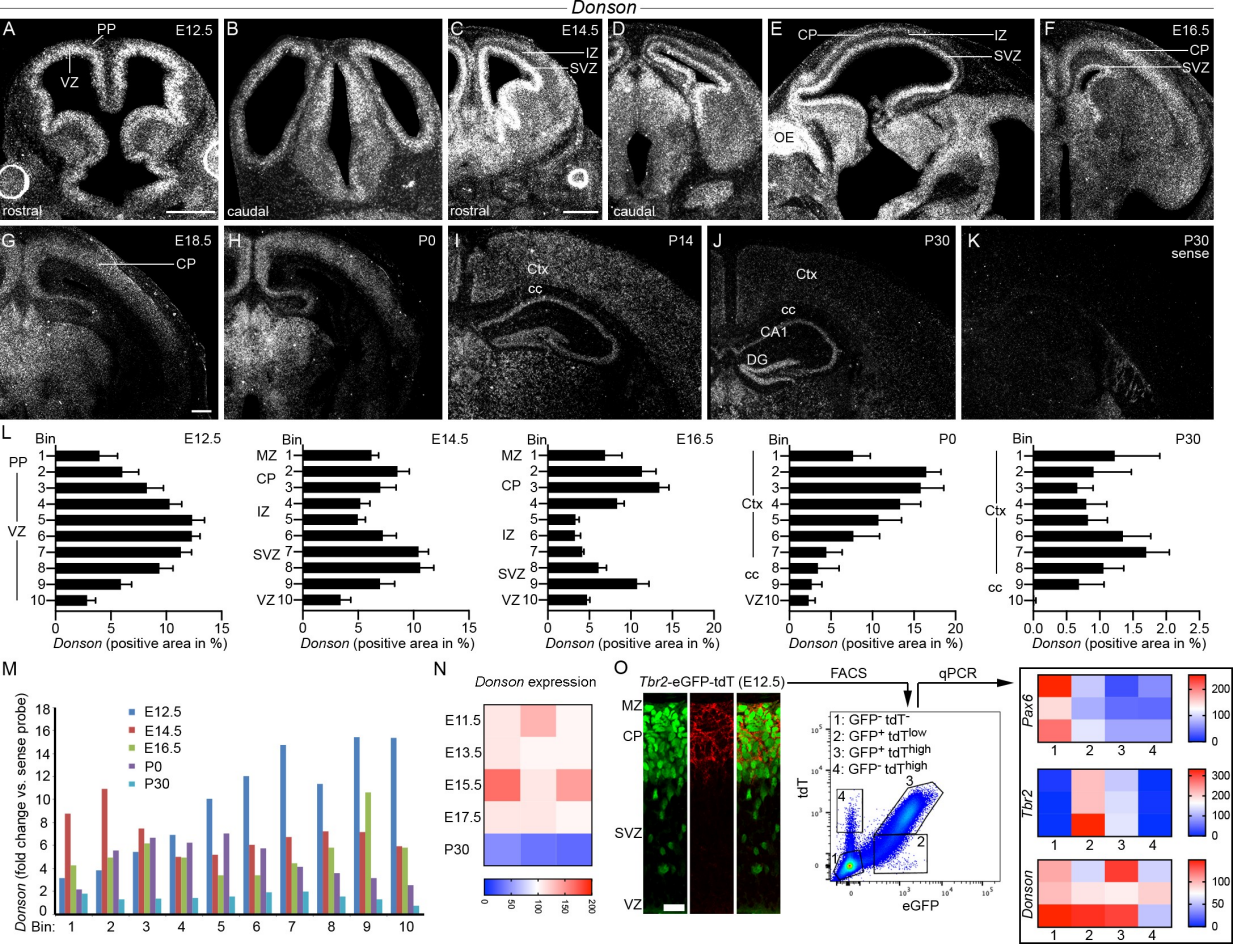
*Donson* expression in the developing telencephalon. (A-J) Darkfield micrographs show *Donson* expression in coronal (a.-d. and f.-j.) and sagittal (e.) sections of the telencephalon at the indicated developmental stages after *in situ* hybridization with a ^35^S-labeled probe. (K) The image demonstrates the signal intensity produced by the sense strand probe (*sense*) at P30. (L) Quantification of the *Donson* hybridization signal in the M1 region of the neocortex after subdividing into 10 equally-sized bins (bin 1 corresponds to the subpial layer). Graphs depict the positive area fraction as mean+SEM. (M) *Donson* hybridization signal in 10 neocortical bins (mean of the positive area fraction) expressed as fold change versus the signal of the *sense* probe. (N) The heat map shows a qPCR analysis of *Donson* expression levels in RNA extracts of dorsal telencephalon (E11.5 –E17.5) and cortex (P30). Values are given as percentage of the overall mean. (O) The confocal image shows fluorescence signals in the E12.5 neocortex for reporter mice expressing nuclear GFP and cell membrane tdT from the *Tbr2* locus. Stable nuclear GFP labels IPC and IPC-derived neurons [22]. The tdT signal emerges later than GFP and labels IPC-derived neurons [22]. The scatter plot demonstrates FACS from E12.5 dorsal telencephalon. Four cell populations were defined using the GFP/ tdT signals of the *Tbr2* reporter. The heat maps show qPCR analysis of *Pax6*, *Tbr2*, and *Donson* in the four populations. Values were first normalized to *Gapdh* and then expressed as percentage of the overall mean of the heat map. Measurements and statistics are summarized in **S1A Table**. Abbreviations: CA1, hippocampal subfield CA1; cc, corpus callosum; CP, cortical plate; Ctx, cerebral cortex; DG, dentate gyrus; IZ, intermediate zone; MZ, marginal zone; OE, olfactory epithelium; PP, preplate, SVZ, subventricular zone, VZ, ventricular zone. Scale bars: 300 μm (a. and c.), 200 μm (g.), 20 μm (o.).

We analyzed the spatial and temporal patterns of *Donson* expression in the developing telencephalon using *in situ* hybridization **(Figs 1A–1M and S1A)**. From E11.5 to E16.5, we observed particularly strong labeling in the proliferation zones of the dorsal and ventral telencephalon **(Figs 1A–1F, 1L, 1M and S1A**), suggesting that *Donson* is present in dorsal progenitors generating cEN and in ventral progenitors generating GABAergic neurons including cIN. At E14.5, the signal within the proliferation zone appeared somewhat stronger in the SVZ than in the VZ (**Fig 1L** and **1M**), suggesting *Donson* might be expressed in IPC. *Donson* was also present in areas populated by post-mitotic neurons (**Fig 1A–1J**). These included the preplate (E12.5), cortical plate (CP: E14.5 to E18.5), marginal zone (MZ: E14.5 to E18.5), forming cortical layers I–VI (E18.5 to P14), hippocampal pyramidal layer (E18.5 to P14), and the subpallial mantle zone (E14.5—E16.5). Areas of white matter, such as the intermediate zone (IZ: E14.5 to E18.5) and the forming corpus callosum (E18.5 to P14), expressed *Donson* at a lower level than the proliferation zones and the neuronal differentiation zones. After P14, the overall signal intensity decreased strongly, but remained above background in areas of grey matter, such as the neocortical layers I–VI, hippocampal pyramidal layer, dentate gyrus granule cell layer, as well as the subependymal and subgranular zones (**Fig 1J–1M**). Higher *Donson* expression at embryonic than at postnatal stages was confirmed using qPCR on RNA extracts from dorsal telencephalon (E11.5 –E17.5) and cerebral cortex (P30) **(Fig 1N)**.

To test whether *Donson* is present in IPC and IPC-derived neurons, we made use of a *Tbr2* reporter expressing nuclear GFP and cell membrane-bound tdT under the control of the *Tbr2* locus [22]. This reporter generates GFP in *Tbr2*^*+*^ IPC and, due to the stability of the nuclear GFP, also in *Tbr2*^*-*^ IPC-derived neurons [22]. In the embryonic cortex, the *Tbr2*-tdT signal emerges later than that of *Tbr2*-GFP [22]. Consequently, tdT is bright in GFP^+^ *Tbr2*-derived neurons and faint in GFP^+^ IPC present in the SVZ (**Fig 1O**). Using FACS on dissociated E12.5 dorsal telencephalon, we isolated an IPC-enriched population characterized by a GFP^+^ tdT^low^ signature, high *Tbr2*, and low *Pax6* expression (*Pax6* is a RGC marker). We further sorted a GFP^+^ tdT^high^ population with low *Tbr2* and absent *Pax6* expression (i.e. IPC-derived neurons). The populations #2 amd #3 **(Figs 1O and S1B)** expressed *Donson* at a similar level as the GFP^-^ tdT^-^ *Pax6*^high^ population #1 (**Fig 1O**). A somewhat lower *Donson* signal was present in population #4, a small cell fraction characterized by a GFP^-^ tdT^high^ *Pax6*^-^ *Tbr2*^-^ signature (**Fig 1O**, presumably early-generated neurons). Collectively, these data demonstrate that *Donson* is highly expressed in the proliferation and differentiation zones of the embryonic dorsal and ventral telencephalon and keeps being expressed at moderate to low levels in the postnatal brain.

### The neuroepithelium of the early pallium depends critically on *Donson*

**Fig 2:**
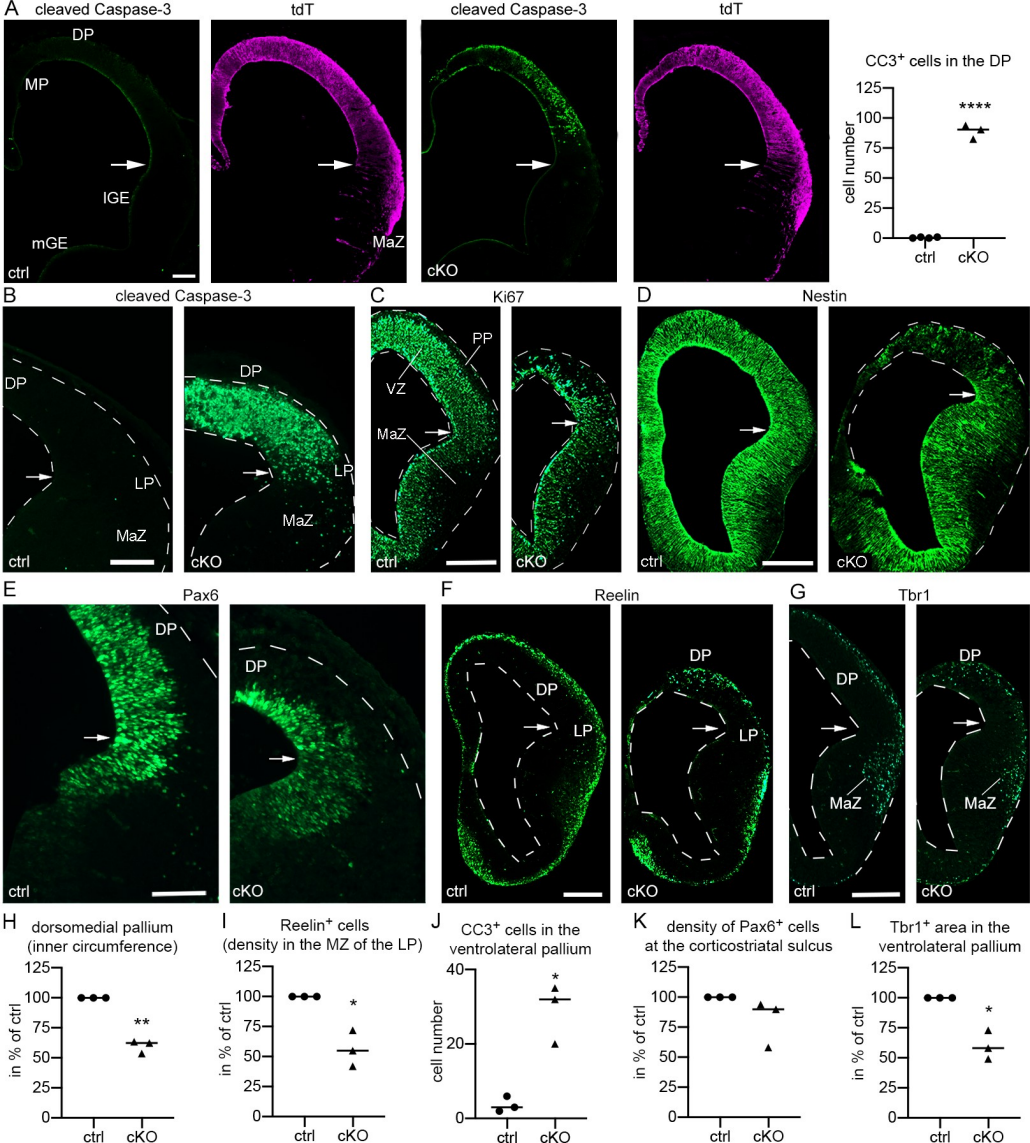
*Donson* deletion in the *Emx1* lineage induces apoptosis in the early pallium. (A-G) Micrographs show rostral coronal brain sections from E11.5 (a.) and E12.5 (b.-g.) control (ctrl) and *Emx1*-cKO (cKO) mice. Arrows identify the corticostriatal sulcus. Mice in (a.) contain a *Rosa26*^CAG-LSL-tdT^ allele. Double immunofluorescences in (a.) identify CC3^+^ apoptotic cells and *Emx1*-Cre labeled tdT^+^ cells. The scatter plot in (a.) shows the number of CC3^+^ cells per 0.015 mm^2^ in the dorsal pallium. Single immunofluorescences in (b.-g.) demonstrate CC3^+^ cells, Ki67^+^ proliferating cells, Nestin^+^ and Pax6^+^ RGC, Reelin^+^ Cajal-Retzius cells, and Tbr1^+^ pallial neurons. (H-L) Scatter plots show the circumference of the dorsomedial pallium as measured along the ventricle boundary from the hem to the corticostriatal sulcus (h.), the number of Reelin^+^ cells in the lateral MZ (i.), the evaluated area is indicated by LP in (f.), the number of CC3^+^ cells ventral to the corticostriatal sulcus (j.), the density of Pax6^+^ RGC in the VZ as measured immediately ventral to the corticostriatal sulcus (k.), and the Tbr1^+^ area ventral to the corticostriatal sulcus (l.). Values in (h., i., k., and l.) are expressed as percentage of the ctrl mean. Measurements and statistics are summarized in **S1B Table**. Abbreviations: DP, LP, and MP, dorsal, lateral, and medial pallium; MaZ, postmitotic mantle zone of the ventrolateral pallium; PP, preplate; VZ, ventricular zone. Scale bars: 30 μm (a. and d.), 120 μm (b., c., e. and f.), 125 μm (g.).

The emerging role of DONSON in microcephaly and its strong expression in the telencephalic proliferation zones prompted us to test whether pallial progenitors require *Donson* function. To this end, we generated *Emx1*^IRES-Cre^; *Donson*^LoxP/LoxP^ conditional knockout mice (*Emx1-*cKO). *Emx1* expression in the mouse dorsal telencephalon starts at E9.5 [23] and *Emx1*-Cre targets nearly every cell of the dorsal, medial and lateral pallia until E12.5 [24]. Since we expected a delay between the onset of Cre production and loss of Donson protein, we began our study at E11.5. Nissl-staining (**S2A Fig**) and the use of a *Rosa26*^CAG-LSL-tdT^ reporter to detect *Emx1*-Cre-labeled pallial neurons **(Fig 2A)** revealed that the pallium of E11.5 *Emx1-*cKO mice was macroscopically intact and of regular size. For further analyses, we subdivided the pallium into a dorsomedial part (i.e. the area from the hem to the corticostriatal sulcus) and a ventrolateral part (i.e. the pallial area from the corticostriatal sulcus to the piriform cortex/ subpallium boundary). At high magnification, we detected numerous cells with an apoptotic morphology in the dorsomedial pallium. Apoptotic cells were more frequent in superficial than in ventricle-facing layers **(S2C Fig)**. Increased apoptosis in the dorsomedial pallium of E11.5 *Emx1-*cKO mice was confirmed by assessing the apoptosis marker cleaved Caspase-3 (CC3) (**Figs 2A and S2D**).

At E12.5, the size of the dorsomedial pallium of *Emx1-*cKOs was markedly reduced compared to that of control littermates **(Figs 2H and S2B)** and CC3^+^ cells were now abundant in all layers of the mutant dorsal pallium **(Fig 2B)**. The massive cell degeneration was associated with a loss of cells expressing the proliferation marker Ki67 and the RGC markers Nestin and Pax6. The signal loss of Ki67 and Pax6 was least severe in the ventricle-facing layers (**Fig 2C–2E**). We next visualized postmitotic neurons by immunostaining E12.5 mice for Tbr1 [25] and Reelin, the latter of which identifies Cajal-Retzius cells originating in the medial pallium, ventral pallium, and septum [26,27]. In the dorsal pallium, Tbr1^+^ and Reelin^+^ neurons were confined to the preplate in controls, but were aberrantly distributed in the VZ in *Emx1*-cKO mice (**Fig 2F** and **2G**). Lateral to the corticostriatal sulcus, where the damage in the neuroepithelium was less severe than dorsally, Reelin^+^ cells exhibited no misplacement, but were reduced by 43% (**Fig 2F** and **2I**). Thus, *Emx1*-Cre-mediated *Donson* deletion triggers massive apoptosis and misplacement of preplate neurons in the early dorsomedial neuroepithelium. The latter effect most likely is a consequence of structural damage, because it is not observed in the less affected lateral pallium.

We then turned to the progenitor domain of the ventrolateral pallium, which is located in the VZ lateral and immediately ventral to the corticostriatal sulcus [28,29]. This region lacked CC3^+^ at E11.5 **(Fig 2A:** arrows**)**, but contained numerous CC3^+^ cells at E12.5 (**Fig 2B:** arrows). The progenitor markers Pax6, Ki67, and Nestin appeared unaltered at the corticostriatal sulcus in E12.5 mutants (**Fig 2C–2E:** arrows).

Postmitotic neurons of the ventrolateral pallium occupy the ventrolateral mantle zone and can be identified by Tbr1 **(Fig 2G:** MaZ**)**. In E12.5 mutants, the Tbr1^+^ area ventral to the corticostriatal sulcus was reduced **(Fig 2G:** MaZ**)**, which was associated with an increase in CC3^+^ cells in the mantle zone **(Fig 2B:** MaZ**)**. These qualitative observations were substantiated by quantifications **(Fig 2J–2L);** see **Methods** for details on the quantification area. Collectively, these findings show that *Emx1*-Cre-mediated *Donson* deletion affects neurogenesis throughout the early pallium, although the effect in the ventrolateral pallium is delayed and less severe than dorsomedially.

### The hippocampus and dorsal neocortex fail to develop in *Emx1*-cKO mice

**Fig 3:**
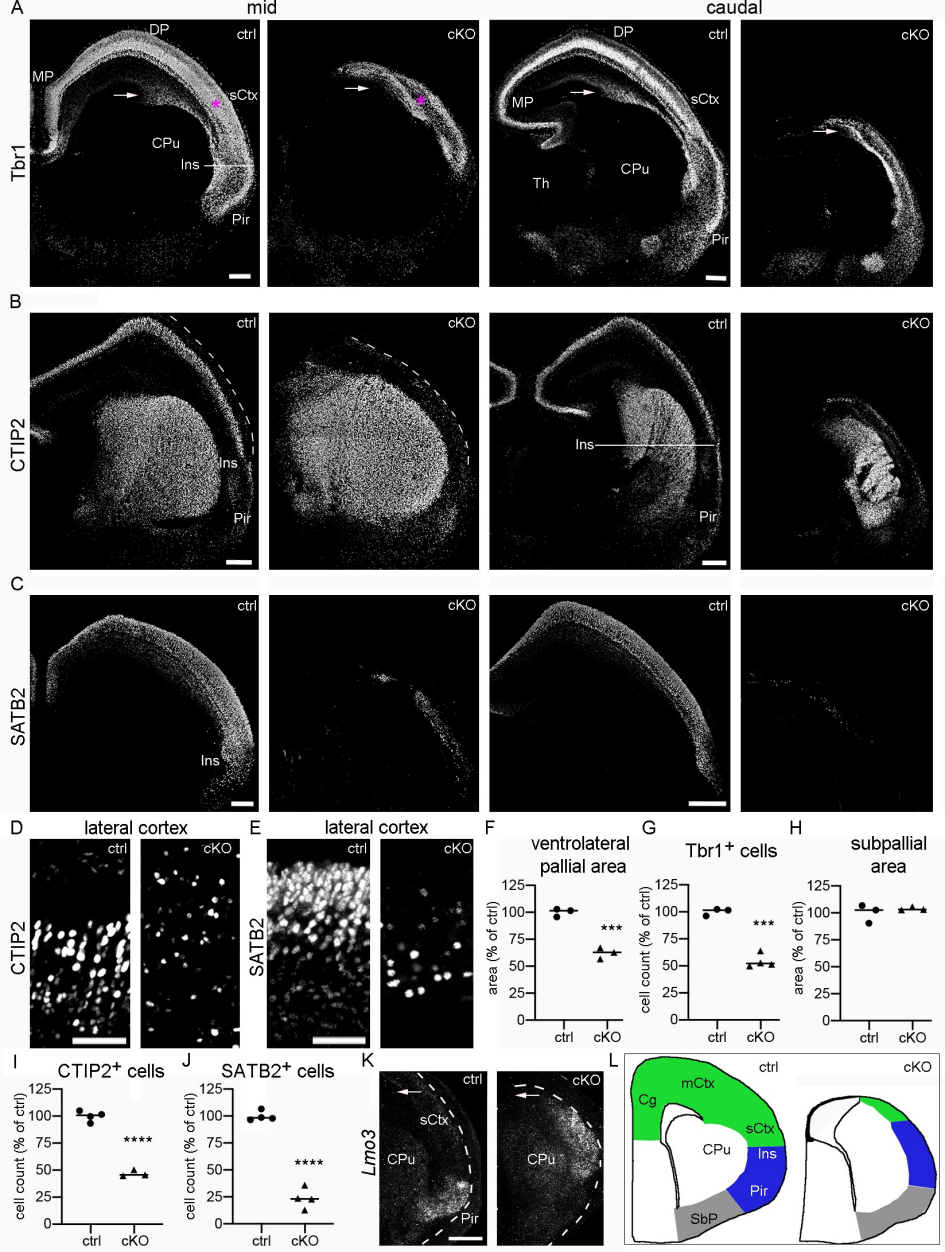
Absence of neocortex is associated with a dorsal shift of piriform cortex in *Emx1*-cKO mice. (A-K) Images and graphs represent E16.5 *Emx1*-cKO (cKO) and control (ctrl) mice. (A-E) Coronal brain sections of cKO and ctrl mice were immunostained for Tbr1, CTIP2, and SATB2. Low magnification images (a.-c.) show mid and caudal sectional planes as indicated. Arrows in (a.) identify the corticostriatal sulcus. High magnification images show CTIP2 (d.) and SATB2 (e.) in the lateral cortex (i.e. in the area identified by asterisks in (a.)). (F-J) Scatter plots show the area of the Tbr1^+^ pallium ventral to the corticostriatal sulcus (f.), the density of Tbr1^+^ cells in the lateral cortex (g.), the area of the subpallium (h.), the density of CTIP2^+^ cells in the lateral cortex (i.), and the density of SATB2^+^ cells in the lateral cortex (j.). Values are expressed as percentage of the ctrl mean. Circles and triangles represent individual E16.5 mice; horizontal lines indicate the median. Measurements and statistics are summarized in **S1C Table**. (K) *Lmo3* is demonstrated at a rostral sectional plane for an E16.5 cKO and a ctrl littermate. Arrows point to the corticostriatal sulcus. (L) Schematic representation of the left rostral telencephalic hemisphere as coronal cross section for E16.5 ctrl and cKO mice. Abbreviations: CPu, caudate-putamen; sCtx, somatosensory cortex; DP and MP, dorsal and medial pallium; Ins, insular cortex; Pir, piriform cortex; SbP, subpallium; Th, thalamus. Scale bars: 200 μm (a.–c.), 50 μm (d. and e.), 200 μm (k.).

We next assessed the dorsomedial pallium at E13.5, when cEN begin to establish the CP. We visualized cEN and the proliferation zone by staining for Tbr1 [25,30] and Ki67, respectively **(S3A–S3E Fig)**. E13.5 mutants exhibited a strikingly increased cortical malformation compared to E12.5 mutants: at mid and caudal sectional planes, the dorsomedial pallium was reduced to a thin sheet of cells that was almost devoid of Tbr1^+^ neurons and Ki67^+^ progenitors. Rostrally, we identified a size-reduced cortex-like structure that contained some Tbr1^+^ neurons in the mantle and Ki67^+^ progenitors in the VZ **(S3A** and **S3D Fig)**. Ablation of the dorsomedial pallium in *Emx1*-cKO mice was confirmed at E16.5 in n>10 *Emx1*-cKO at mid and caudal planes **(Figs 3A and S3H)**. Rostrally, the cortex of E16.5 mutants contained some Tbr1 neurons **(S3F Fig)**, but lacked a CP **(S3H Fig)**. Collectively, these findings show that the hippocampus and dorsal neocortex fail to develop in *Emx1*-cKO mice.

### The lateral neocortex is replaced by paleocortex in *Emx1*-cKO mice

We then turned our attention to the ventrolateral pallium, which includes the somatosensory cortex (lateral), the insular cortex/ claustrum complex (ventral to the somatosensory cortex), and the piriform cortex/ endopiriform nucleus (ventral) **(Fig 3A)**. We focused on E16.5, when cortical sub-regions can be differentiated with specific markers. Using Tbr1 as a pan-pallial marker, we found that the ventrolateral pallium (i.e. the Tbr1^+^ area ventral to the corticostriatal sulcus) was reduced by 38% in *Emx1*-cKO mice (**Fig 3A** and **3F**), which recapitulates our finding for the ventrolateral pallium at E12.5 **(Fig 2L)**. The cell density of Tbr1^+^ neurons was quantified in the lateral cortex (the area labeled with asterisks in **Fig 3A**), showing a 45% reduction in mutants (**Fig 3G**). We further noticed that the ventral boundary of the Tbr1^+^ region (i.e. the ventral boundary of the piriform cortex) was shifted dorsally in E16.5 *Emx1*-cKOs (**Fig 3A**). In addition, the Tbr1 pattern of the mutant lateral cortex resembled vaguely that of the control piriform cortex (i.e. the Tbr1 signal was high in superficial layers) (**Fig 3A**). These observations and the finding that the mutant lateral cortex lacked a CP (**S3H Fig**) prompted the hypothesis that meso- and allocortical structures, such as the insular and piriform cortices, might be present in *Emx1*-cKO mice, where normally the lateral neocortex is localized. To test this, we stained for CTIP2 and SATB2. In the neocortex, CTIP2 is highly expressed in subcortically projecting deep layer neurons and sparsely expressed in upper layer neurons, the inverse expression pattern being present in the insular and piriform cortices [31]. Accordingly, CTIP2 readily permitted identification of the boundary of the lateral neocortex and the insular cortex in controls (**Fig 3B**). The mutant cortex lacked high CTIP2 signal in deep layers. Instead, it contained few CTIP2^+^ neurons dispersed across layers (**Fig 3B, 3D** and **3I**). SATB2 is expressed in postmitotic neocortical neurons that are destined for upper layers, project via the corpus callosum and are generated late (laterally, SATB2^+^ neurons originate mainly at E14.5 and E15.5) [32–34]. We observed abundant SATB2 signal in upper layers of the control lateral cortex as expected, but only few SATB2^+^ neurons in the mutant lateral cortex (**Fig 3C, 3E** and **3J**). Next, we analyzed the pattern of *Lmo3*, a paleocortex marker highly expressed in the E16.5 piriform cortex [29,35,36]. The *Lmo3*^+^ domain was shifted dorsally in *Emx1*-cKOs, thus replacing large parts of the lateral neocortex (**Fig 3K**). Given this dorsal shift of the piriform cortex, we examined the localization of the olfactory tract, a main afference of the piriform cortex. In *Emx1*-Cre mice containing a *Rosa26*^CAG-LSL-tdT^ allele, the olfactory tract can readily be identified by its position at the surface of the piriform cortex and its tdT-signal which emerges from tdT^+^ axons of *Emx1*-Cre-expressing mitral cells in the olfactory bulb [24]. In *Emx1*-cKO mice, the olfactory tract and the ventral boundary of the tdT^+^ pallium were shifted dorsally **(S3G Fig)**. Claustrum and endopiriform nucleus were not discernable in E16.5 *Emx1*-cKO mice. Next, we analyzed the subpallium (i.e. the area ventral to the Tbr1^+^ piriform cortex). In the mutants, this region was enlarged in dorsal direction, but was also narrower than in controls. This was associated with an enlargement of the ventral subarachnoid space at mid and caudal planes **(S3H Fig)**. The size of the subpallial area was not altered **(Fig 3H)**.

Finally, we performed a BrdU pulse-chase experiment to test whether the still remaining cortex in *Emx1*-cKO mice is generated early like the piriform cortex or late like the lateral neocortex [37,38]. To this end, we applied BrdU to pregnant females at E11.5 and immunostained cortices of the embryos for BrdU at E16.5. In our evaluations, we focused on heavily BrdU-labeled cells. First, we examined the lateral neocortex and the corresponding region in mutants as indicated by arrows (**S3H Fig)**. In controls, this region contained few BrdU^+^ cells in early-generated deep layers, whereas in mutants, it harbored numerous BrdU^+^ cells dispersed across all layers **(****S3I** and **S3K Fig****)**. We then turned to the ventral boundary of the pallium (i.e. the boundary of piriform cortex and subpallium). Here, mutants and controls exhibited numerous BrdU^+^ cells dispersed across all layers, the total number being somewhat lower in mutants **(****S3J** and **S3K Fig****)**. Notably, the ventral boundary of the heavily BrdU-labeled piriform cortex was shifted dorsally in the mutants. This BrdU-labeling study shows that the cortical region that normally contains the late-generated lateral neocortex contains an early-generated cortex in *Emx1*-cKO mice. Collectively, these findings suggest that the lateral neocortex is largely absent and replaced by an early-generated piriform-like cortex in E16.5 *Emx1*-cKO mice **(Fig 3L)**.

### The lateral cortical stream (LCS) is absent in E16.5 *Emx1*-cKO mice

**Fig 4:**
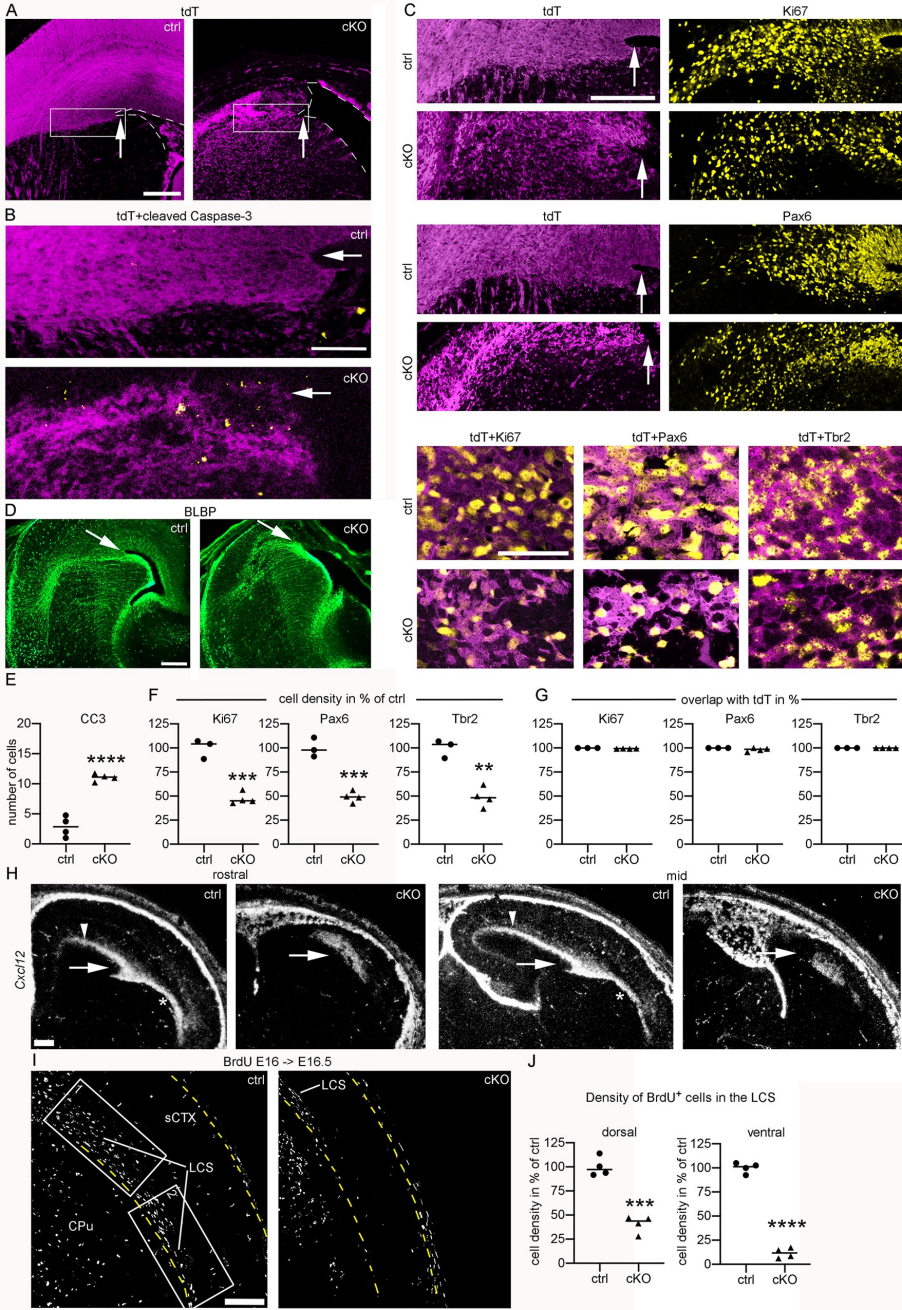
The LCS stream is absent in E16.5 *Emx1*-cKO mice. (A-J) Images and graphs represent E16.5 *Emx1*-cKO (cKO) and control (ctrl) mice. (A-C) Confocal images show coronal brain sections of mice carrying a *Rosa26*^CAG-LSL-tdT^ allele. Sections were immunostained for tdT, CC3, Ki67, Pax6, and Tbr2 as indicated. Arrows indicate the corticostriatal sulcus. Rectangles in (a.) indicate the region of interest for (b.) and (c.). Note increased number of CC3^+^ cells lateral and ventral to the corticostriatal sulcus in the cKO shown in (b.). Images in (c.) represent separate channels at low magnification (upper panel) and overlays at high magnification (lower panel). (D) Epifluorescence images show immunostaining for BLBP. Arrows indicate the corticostriatal sulcus. (E-G) Scatter plots show the number of CC3^+^ cells (e.), the density of Ki67^+^, Pax6^+^, and Tbr2^+^ cells (f.) as well as the overlap of Ki67, Pax6, and Tbr2 with tdT (g.). Measurements were performed in the tdT^+^ progenitor domain lateral and ventral to the corticostriatal sulcus highlighted in (a.). (H) Darkfield micrographs of coronal brain sections demonstrate *Cxcl12* in the pallium of ctrl and cKO mice at a rostral and at a mid sectional plane. Arrows indicate the corticostriatal sulcus, arrowheads the *Cxcl12*^+^ SVZ, and asterisks the LCS. (I) Confocal images demonstrate BrdU^+^ cells in E16.5 mice receiving BrdU on E16. The LCS was photographed immediately ventral to the corticostriatal sulcus. Rectangles #1 and #2 represent dorsal (#1) and ventral (#2) parts of the LCS. (J) Scatter plots show the density of BrdU^+^ cells in regions #1 and #2. Horizontal lines indicate the median. Measurements and statistics are summarized in **S1D Table**. Abbreviations: CPu, caudate-putamen; LCS, lateral cortical stream; sCTX, somatosensory cortex. Scale bars: 180 μm (a.), 140 μm (b.), 180 μm (c. upper panel), 50 μm (c. lower panel), 200 μm (d. and h.), 100 μm (i.).

Having observed that the progenitor number next to the corticostriatal sulcus is unchanged in E12.5 *Emx1*-cKO mice (**Fig 2E** and **2K**), we examined this region again at E16.5 **(Fig 4);** the region of interest is indicated (**Fig 4A)**. Compared to controls, *Emx1*-cKO mice exhibited an increased number of CC3^+^ cells and a ≈50% reduction in the density of Ki67^+^ cells, Pax6^+^ RGC, and Tbr2^+^ IPC in this region (**Fig 4B, 4C, 4E** and **4F**). In control and mutant mice, virtually all progenitors of the examined region exhibited tdT fluorescence from the *Rosa26*^CAG-LSL-tdT^ reporter (**Fig 4C** and **4G**), indicating they were of pallial origin.

Numerous progenitors and neurons that emerge next to the corticostriatal sulcus migrate along radial glia fibers in ventrolateral direction, thereby establishing the LCS, which provides excitatory neurons to pallial regions located ventral to the corticostriatal sulcus [28]. By staining for brain lipid-binding protein (BLBP) we found that RGC originating at the corticostriatal sulcus were severely perturbed in E16.5 *Emx1*-cKO mice: the cells were disorganized and failed to reach the ventral pallial region **(Fig 4D)**. This prompted the hypothesis that the LCS might be defective in *Emx1*-cKO mice. To test this, we hybridized for *Cxcl12*, which is expressed in Tbr2^+^ IPC in the SVZ of the dorsal pallium **(Fig 4H:** arrowheads**)** and along the LCS **(Fig 4H:** asterisks**)** [39–41]. In E16.5 *Emx1*-cKO mice, intracortical *Cxcl12* expression was reduced to a small area lateral to the corticostriatal sulcus **(Fig 4H:** arrows**)** and was absent along the ventral parts of the LCS **(Fig 4H)**. We next labeled proliferating cells by administering BrdU to pregnant females at E16. In E16.5 controls, BrdU-labeling was present in the dorsal part of the LCS (i.e. in close proximity to the corticostriatal sulcus) and in the ventral part of the LCS (**Fig 4I** and **4J**). In *Emx1*-cKO littermates, BrdU^+^ cells were reduced by 59% in the dorsal part of the LCS and were virtually absent in the ventral part of the LCS (**Fig 4I–4J**). Collectively, our findings in E12.5 and E16.5 *Emx1*-cKO mice indicate that a large proportion of the progenitors that establish the ventrolateral pallium degenerate between E12.5 and E16.5. Furthermore, we show that the LCS, which provides the ventrolateral pallium with progenitors and neurons, is absent in *Emx1*-cKO mice, probably because progenitors fail to migrate along the perturbed radial glia scaffold and are retained in proximity to the corticostriatal sulcus.

### cIN accumulate in the ventrolateral cortex of *Emx1*-cKO mice

Next, we examined cIN in the cortex of *Emx1*-cKO mice. We hybridized for *Lhx6*, which identifies mGE-derived cIN [42], and for *Reelin*, which, at E16.5, labels diverse populations of cIN and Cajal-Retzius cells [12,24,26]. Both transcripts were not detected in the rudimentary dorsomedial pallium of E16.5 *Emx1*-cKO mice **(S4A** and **S4B Fig)**. In the ventrolateral pallium, the number of labeled cells was increased for both markers (**S1A–S1E Fig**), suggesting that cIN that failed to invade dorsomedial regions accumulated ventrolaterally. This was confirmed using a 5HT3-eGFP transgene, which identifies cIN originating in the cGE [43] (**S4F Fig**).

### *Tbr2*-Cre-mediated *Donson* deletion in IPC does not induce cortical malformations

Given that *Emx1*-cKO mice contain apoptotic cells in postmitotic zones, such as the preplate/ MZ of the dorsal pallium and the mantle zone of the ventrolateral pallium, we asked whether *Donson* deletion in nascent neurons would induce apoptosis. To approach this question, we employed *Tbr2*-Cre, which targets IPC generating up to 67% of cortical neurons [10]. We reasoned that, due to the delay between Cre-mediated *Donson* deletion and loss of pre-formed Donson protein, *Tbr2*-Cre should have no or only small effects on IPC as these cells transition quickly into neurons. With *Tbr2* being expressed in IPC from E11.5 onward [44], we examined *Tbr2*-Cre; *Donson*^LoxP/LoxP^ (*Tbr2*-cKO) mice at E14.5, E16.5, and P0. The mutants did not show increased CC3 staining in the cortex and exhibited a neocortex and hippocampus of regular size (**S5 Fig**). Under the assumption that Donson protein is depleted in IPC-derived neurons of *Tbr2*-cKOs until P0, these findings indicate that, other than proliferating RGC, postmitotic neurons do not require Donson for survival.

### *Donson* is essential for progenitors generating cIN

**Fig 5:**
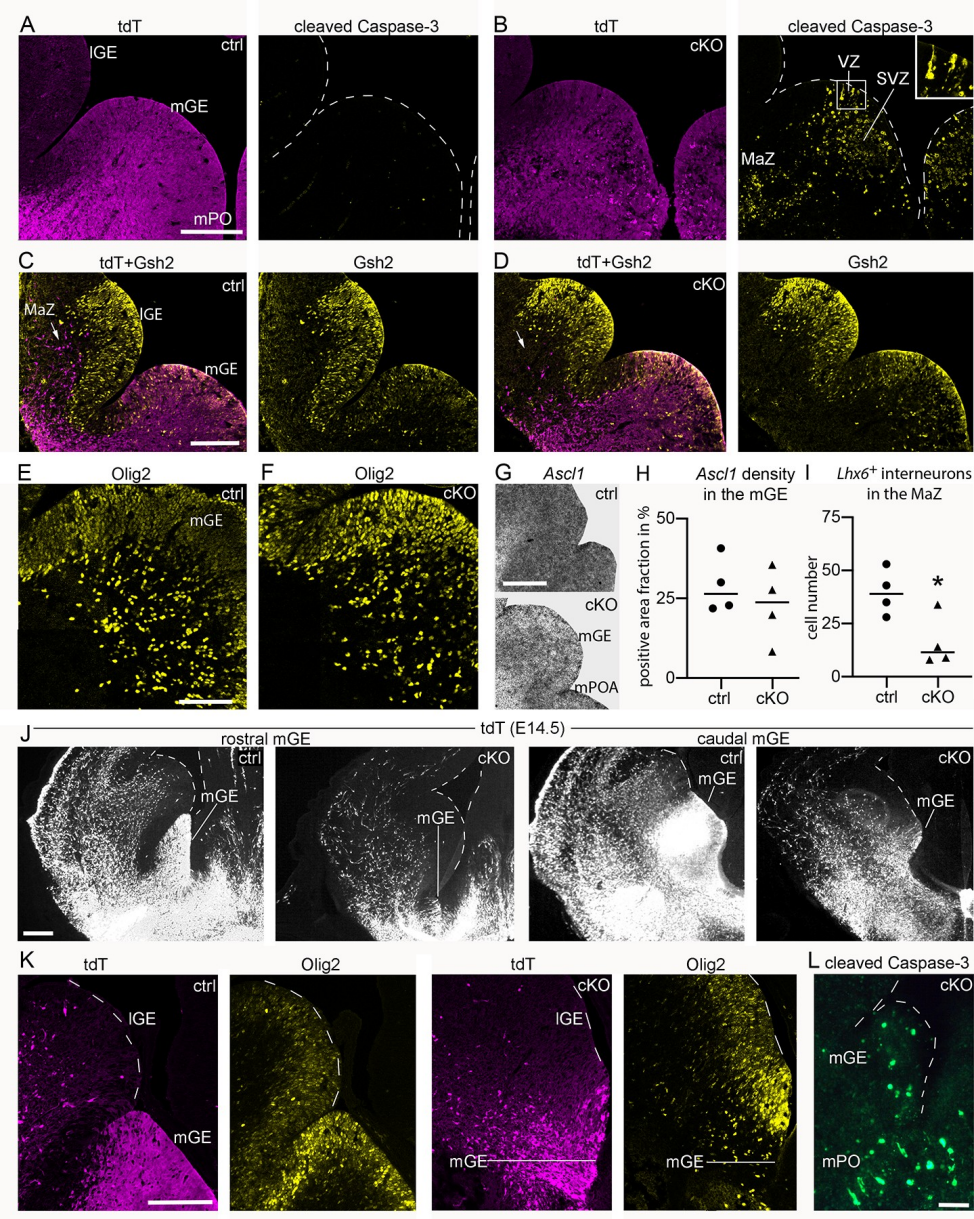
*Nkx2-1*-Cre-mediated deletion of *Donson* induces apoptosis in the mGE. (A-L) Images and graphs represent *Nkx2*.*1*-cKO mice (cKO) and control (ctrl) littermates at E12.5 (a.-i.) and E14.5 (j.-l.). Mice carried a *Rosa26*^CAG-LSL-tdT^ allele. (A,B) Confocal images demonstrate native tdT and immunostaining for CC3 in the mGE and mPO of ctrl and cKO mice. The inset in (b.) shows apoptotic cells in the VZ at high magnification. (C,D) Dual immunofluorescences demonstrate tdT and Gsh2 in the subpallium at E12.5. Note that tdT^+^ cells are missing in the subpallial mantle zone of the cKO (arrows). (E,F), Immunostaining for Olig2 in the mGE at E12.5. (G) Micrographs demonstrate *Ascl1* in the E12.5 mGE and mPO after *in situ* hybridization with a ^35^S-labeled probe (dark signals). (H,I) Scatter plots show quantifications of *Ascl1* expression in the VZ of the mGE (h.) and the number of *Lhx6*^+^ cells in the mantle zone lateral to the lGE (i.); see arrow in (c.) for the evaluated region. Circles and triangles represent individual mice, horizontal lines represent the median. Measurements and statistics are summarized in **S1E Table**. (J) Confocal images demonstrate two sectional planes of the mGE at E14.5. The mGE is identified by strong tdT signal in the VZ and SVZ. (K) Confocal images show double immunofluorescences for tdT and Olig2 in the mGE at E14.5. (L) Epifluorescence image demonstrates CC3 in the mGE/ mPO area of an E14.5 cKO. Abbreviations: lGE and mGE, medial and lateral ganglionic eminence; mPO, medial preoptic area; MaZ, subpallial mantle zone; SVZ, subventricular zone; VZ, ventricular zone. Scale bars: 200 μm (a. and c.), 100 μm (e.), 200 μm (g.), 180 μm (j.), 150 μm (k.), 60 (l.) μm.

Having established that *Donson* is expressed in the progenitor domain of the ventral telencephalon, we asked whether lack of Donson affects the generation of cIN. We focused on cIN originating in the mGE and mPO, which can be efficiently targeted at the progenitor stage using *Nkx2*.*1*-Cre [45]. *Nkx2*.*1* is highly expressed in the mGE and mPO starting at E9.5 [46,47], and *Nkx2*.*1*-Cre generates robust recombination in these regions at E12.5 [45]. We thus began our assessment of *Nkx2*.*1*-Cre; *Donson*^LoxP/LoxP^ (*Nkx2*.*1*-cKO) mice at E12.5. Serial section analysis showed that although the mGE and mPO appeared macroscopically intact, they contained an abundance of CC3^+^ cells (**Fig 5A** and **5B**). These were mainly present in the SVZ and the postmitotic mantle zone and were somewhat less frequent in the VZ (**Fig 5A** and **5B**). Examination of the mGE using Gsh2, Olig2 and *Ascl1* as markers for subpallial RGC and progenitors [48,49] revealed that the majority of these progenitors were still present in E12.5 *Nkx2*.*1*-cKO mice **(Fig 5C–5H)**. This was in sharp contrast to *Nkx2*.*1*-labeled tdT^+^ cells in the mantle zone lateral to the lGE: this cell population was massively reduced in E12.5 *Nkx2*.*1*-cKO mice (arrows in **Fig 5C** and **5D**). Since *Nkx2*.*1*-derived cells in the lGE mantle zone of E12.5 mice are considered as precursors of cIN [45], this reduction points to a defect in the generation of cIN. Consistently, E12.5 *Nkx2*.*1*-cKO mice exhibited a 60% reduction in the number of *Lhx6*^*+*^ cells in the mantle zone lateral to the lGE **(Fig 5I)**.

Next, we performed a serial section analysis of the ventral telencephalon at E14.5. While the mGE could readily be identified as an anatomical entity in E14.5 controls, it was barely detectable in *Nkx2*.*1*-cKO littermates (n = 5 mice each) **(Fig 5J)**. Furthermore, the Olig2^high^ progenitor domain, which is characteristic of the mGE [49], was almost absent in the ventral telencephalon of the mutants **(Fig 5K)**. The still remaining parts of the mutant mGE and mPO contained numerous CC3^+^ cells in the VZ and SVZ **(Fig 5L)**, indicating still ongoing apoptosis of progenitors.

**Fig 6:**
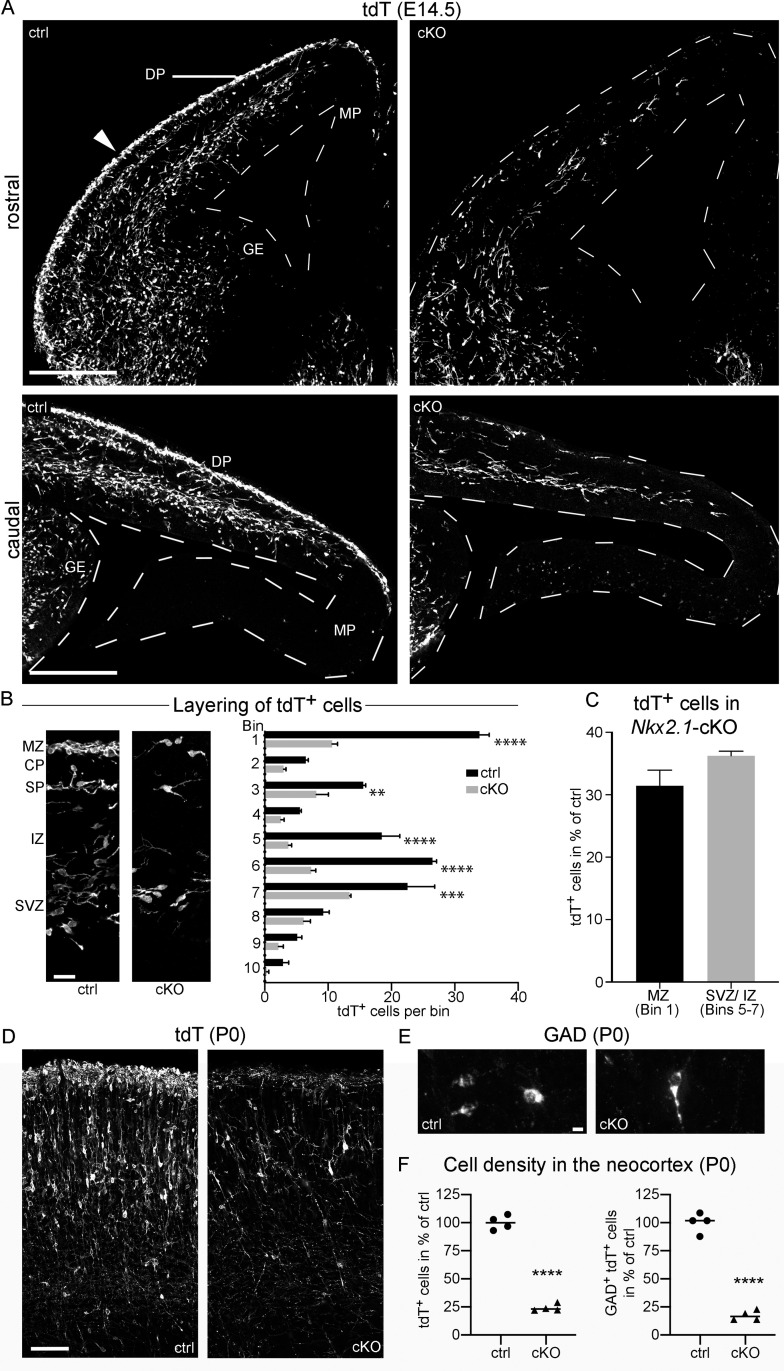
The number of mGE-derived cIN is reduced in *Nkx2*.*1*-cKO mice. (A-F) Images and graphs compare *Nkx2*.*1*-cKO (cKO) mice and control (ctrl) littermates at E14.5 (a.-c.) and P0 (d.-f.). Mice carried a *Rosa*26^CAG-LSL-tdT^ allele. (A) Confocal images demonstrate tdT^+^ cells in the telencephalon at rostral and caudal sectional plains as indicated. Note reduced number of tdT^+^ cells in the cortex of the cKO. (B) High magnifications of the neocortex demonstrate that the layering of tdT^+^ cells in the MZ, SP, and SVZ/ IZ is preserved in cKO mice. The graph shows the number of tdT^+^ cells per bin for the neocortex as mean+SEM. Bin 1 corresponds to the MZ and bin 10 to the VZ. The arrowhead in (a.) identifies the neocortical area assessed in (b.). (C) The graph compares the number of tdT^+^ cells present in the MZ (bin 1) and SVZ/ lower IZ (bins 5+6+7) of cKO mice; data correspond to those in (b.). Values are presented as mean+SEM after normalization with the control mean. Note that the cell number is reduced to a similar extent in the MZ and SVZ/ IZ. (D) tdT signal in the M1 region of the neocortex at P0. (E) GAD immunofluorescence in the M1 region of the P0 neocortex. (F) Scatter plots show the densities of tdT^+^ and of tdT^+^ GAD^+^ cells in the M1 region of the neocortex as percentage of the control mean. Circles and triangles represent individual mice, horizontal lines represent the median. Measurements and statistics are summarized in **S1F Table**. Abbreviations: DP and MP, dorsal and medial pallium; GE, ganglionic eminence. Scale bars: 220 μm (a.), 25 μm (b.), 110 (d.), 6 μm (e.).

To substantiate the assumption that the production of cIN is affected in *Nkx2*.*1*-cKO mice, we assessed tdT^+^ cells in the neocortex at E14.5, when numerous *Nkx2*.*1*-derived cIN migrate within the cortex [45]. In *Nkx2*.*1*-cKO mice, the number of these cells was reduced by 67% (**S2 Table;** the quantified area is indicated by an arrowhead in **Fig 6A**). Oligodendrocyte precursors (OPC) represent a second cell population originating from *Nkx2*.*1*^+^ progenitors in the mGE. At E14.5, telencephalic OPC are thought to be exclusively mGE-derived and have not invaded the cortex yet [50]. We quantified Olig2^+^ cells in the mantle zone of the lGE as these cells correspond to OPC [49]. This showed a 64% reduction in *Nkx2*.*1*-cKO mice (**S3 Table**). Collectively, our findings establish that *Nkx2*.*1*-Cre-mediated *Donson* deletion ablates large parts of the progenitor domain of the mGE/ mPO between E12.5 and E14.5. Furthermore, the bulk of mGE-derived cIN and mGE-derived OPC are absent in E14.5 *Nkx2*.*1*-cKO mice.

To answer whether *Donson* deficiency affects the migration of cIN, we assessed the layering of tdT^+^ cells in the cortex at E14.5, when mGE-derived cIN are organized in distinct migration streams [45]. We focused on the neocortical region next to the corticostriatal sulcus (arrows in **Fig 6A**). In both, mutants and controls, tdT^+^ cells were mainly present in the MZ (bin 1), SVZ/ IZ (bins 5–7), and to a lesser extent in the subplate (bin 3) (**Fig 6B**). The number of tdT^+^ cells in the mutants was reduced to a similar extent in the MZ and the SVZ/ IZ (**Fig 6C**), suggesting that the allocation of cIN to the distinct tangential migration streams is largely intact in *Nkx2*.*1*-cKO mice. More medially, tdT^+^ cells were present only in the SVZ/ IZ in *Nkx2*.*1*-cKOs, whereas they populated the SVZ/ IZ and the MZ in controls **(Fig 6A)**. We assume that this difference is because more cIN enter the SVZ/ IZ than the MZ, which leads to an earlier depletion of cIN along the lateromedial migration path in the MZ than in the SVZ/ IZ.

Finally, we assessed cortices of neonatal *Nkx2*.*1*-cKO mice. In the M1 region, the mutants exhibited a 76% reduction of tdT^+^ cells and a 82% reduction of GAD^+^ tdT^+^ cells compared to control littermates **(Fig 6D–6F)**, indicating that the early loss of cIN is not compensated in *Nkx2*.*1*-cKO mice.

## Discussion

Although a wealth of data shows that *DONSON* mutations lead to PM, skeletal abnormalities, and probably hematopoiesis defects [14,16–21], consequences of *DONSON* loss of function have not been assessed in animal models. Here, we identify *Donson* expression in the major proliferation zones of the mouse telencephalon. *Donson* deletion in telencephalic progenitors using *Emx1-Cre* and *Nkx2*.*1-*Cre induces massive apoptosis in the early dorsomedial pallium and mGE/ mPO, respectively. Whilst in both pallium and mGE single apoptotic progenitors are in direct contact with the ventricle, the bulk of apoptotic cells are remote from the ventricle: in the early pallium, apoptotic cells reside in superficial and mid cortical layers, and in the mGE they occupy the mantle zone and SVZ. Given the spatial organization of progenitors and neurons in the cortex and mGE [48,51], this suggests that mainly S-phase and nascent postmitotic cells become apoptotic upon *Emx1*- and *Nkx2*.*1*-Cre-mediated *Donson* deletion. At later embryonic stages, *Donson* mutants fail to develop the hippocampus, neocortex, LCS (*Emx1*-cKO), and up to 76% of mGE-derived cIN (*Nkx2*.*1*-cKO). This is in sharp contrast to *Tbr2*-cKO mice. Although *Tbr2*-Cre is active in the cortex from E11.5 onward and targets up to 67% of cortical excitatory neurons [10], *Tbr2*-cKOs exhibit no increase in cortical CC3, and, until P0, no apparent cortical malformations. Whilst this argues against a crucial function of Donson in the survival of young neurons, it does not exclude the possibility that IPC require Donson for mitosis, because already existing Donson protein might permit IPC to replicate despite undergoing *Tbr2*-Cre-mediated *Donson* deletion. Collectively, our findings indicate that neural progenitors in the dorsomedial pallium and mGE/mPO depend critically on Donson function, which is in keeping with DONSON being essential for proliferating cells by acting as a replisome component protecting the replication fork and telomere ends [15,16].

We further identify an abnormal ventrolateral pallium in *Emx1*-cKO mice (i.e. cortical structures ventral to the corticostriatal sulcus): it is smaller than the corresponding region in controls and exhibits an abnormal high density of cIN. The apoptosis onset in the progenitor domain of the ventrolateral pallium is delayed compared to that of the dorsomedial pallium. We therefore assume that substantial parts of the mutant ventrolateral pallium are formed before the Donson protein is depleted in the relevant progenitors. Using BrdU pulse-chase, we find that the mutant lateral cortex develops before the corresponding region of controls (i.e. the lateral neocortex) [38]. This suggests that an early-generated cortex is present at the place of the late-generated lateral neocortex in *Emx1*-cKO mice. In support of this, the mutant lateral cortex lacks the cortical plate and the neocortical patterns of SATB2 and CTIP2, but expresses the piriform cortex marker *Lmo3*. Furthermore, the olfactory tract (the main piriform afference) is shifted dorsally along with the ventral boundary of the piriform cortex as defined by Tbr1 and *Emx1*-Cre-labeling. This phenotype is reminiscent of the *Gli3*^-/-^ brain, where an *Lmo3*^+^ paleocortex is specified next to a rudimentary dorsomedial pallium [36]. It also corresponds to *Emx1*-Cre; *Lhx2*^LoxP/LoxP^ mice, where a piriform-like cortex develops at the position of the lateral neocortex [52]. In the latter model, neocortical progenitors are thought to become respecified to generate paleocortex [52]. This is distinct from our findings in *Emx1*-cKO mice, where the early-generated paleocortex shifts dorsally as the neocortex degenerates.

Our finding that progenitors of the neocortex are particularly sensitive to perturbed Donson function is consistent with the observation that hypotrophy of the neocortex can be associated with age-appropriate morphology of other brain structures in humans with biallelic *DONSON* mutations [18]. Our finding that progenitors of cIN also depend on *Donson* raises the possibility that the balance of cIN and cEN might be altered in humans with *DONSON*-related developmental defects.

Ablation of a progenitor subpopulation is an important experimental approach for defining the function of the targeted cell lineage. Our findings with *Emx1*-Cre and *Nkx2*.*1*-Cre suggest that targeting *Donson* with a suitable Cre-driver is a feasible way to kill off progenitors and nascent postmitotic cells. In this context, it should be noted that we observed high *Donson* expression throughout the E12.5 prosencephalon, mesencephalon, and myelencephalon, suggesting that Donson might be important for a broad spectrum of neural progenitor subpopulations. Given that DONSON is overexpressed in advanced dedifferentiated carcinomas [53], the gene might also be targeted for therapeutic purposes.

In summary, we established that *Donson* is essential for diverse telencephalic progenitor populations. Our findings suggest that PM in individuals with DONSON mutations results from apoptosis of early pallial RGC and may also be associated with defects in the production of cIN.

## Methods

Mouse husbandry was in accordance with institutional and EU or national guidelines for animal use, approved by the competent authority (Thüringer Landesamt für Verbraucherschutz, TLV), and supervised by the institutional veterinarians. Animal procedures were performed in adherence to our project licenses issued by the federal state Thueringen (TLV administrative authorization number UKJ-17-018 and UKJ-20-008).

### Mice

Mice were kept on C57BL/6j background under temperature-controlled conditions (20–24°C) with a 12 h dark-light cycle and free access to food and water. Noon of the day after mating was considered E0.5. Animals were used irrespective of sex and allocated to experimental groups only according to their genotype. A *Donson*^tm1a(EUCOMM)Wtsi^ allele was produced as part of the European Conditional Mouse Mutagenesis Program (EUCOMM) and the International Knockout Mouse Consortium. The conditional *Donson*^LoxP^ allele (*Donson*^tm1c^) was generated by breeding *Donson*^tm1a^ mice with mice expressing a ubiquitous Flp recombinase. *Donson*^LoxP^ was verified using Southern blot and PCR. *Emx1*-Cre, *Nkx2*.*1*-Cre, *Tbr2*-Cre, Ai14 *Rosa26*^CAG-LSL-tdT^, and *Tbr2* reporter mice were described [22,24,45,54,55]. Mice containing one of the Cre-alleles and two *Donson*^LoxP^ alleles were defined as conditional knockouts (*Emx1*-cKO; *Nkx2*.*1*-cKO, *Tbr2*-cKO). Control cohorts consisted of littermates lacking the Cre allele or littermates with one or two *Donson*^Wt^ alleles. For BrdU labeling, pregnant females received 50 mg/ kg BrdU (i.p.).

### *In situ* hybridization and quantification of hybridization signals

*In situ* hybridization was carried out as described using ^35^S-labeled or digoxigenin-labeled riboprobes [56,57]. The *Donson* probe corresponded to the *Donson* coding sequence and was controlled using the sense strand probe. Probes for *Lmo3* and *Ascl1* correspond to probes in the Allen Mouse Brain Atlas [58]. Probes for *Reelin* and *Lhx6* have been described [40]. Quantitative analysis of hybridization signals was performed using ImageJ. Briefly, brightfield micrographs were captured with Axio Imager A1 (Zeiss) connected to ProgRes C5 camera (Jenoptik). For quantification of *Donson*, the M1 region of the neocortex was automatically subdivided into 10 equally sized horizontal bins with bin 1 corresponding to the subpial layer and bin 10 to the VZ (E11.5 –P0) or corpus callosum (P30). A uniform threshold was applied before measuring the positive area fraction in binarized images. The signal of the sense probe was subtracted. To calculated fold change of *antisense* versus *sense*, values of at least 3 *antisense* specimens were averaged and divided by the value of an age-matched *sense* specimen. The *Ascl1*^*+*^ area fraction was determined after placing a defined region of interest in the ventricular zone of the mGE and setting a uniform threshold. *Lhx6* and *Reelin* signals (positive area fraction) were quantified in the E16.5 lateral cortex after generating 10 bins and setting a uniform threshold.

### Immunohistology

Embryos were fixed in 4% PFA, PBS, pH7.4. Tissue was cryoprotected using 30% sucrose in TPBS buffer (10 mmol/l Tris, pH 7.4, 10 mmol/l phosphate, 155 mmol/l NaCl). Sections were either processed as 40 μm free floating coronal cryosections or as 20 μm slide-mounted coronal cryosections. Sections were incubated for 30 min in 50% methanol, TPBS before blocking with 3% BSA, 0.3% Triton X-100 in TPBS for 1 h. Primary antibody was applied over night in working buffer (1% BSA, 0.3% Triton X-100 in TPBS). Secondary antibody was applied at 1:400 in working buffer for 2 h at room temperature. Washing steps were performed with 0.3% Triton X-100 in TPBS. Primary antibodies, dilutions, and the use of signal amplification are listed in **S5 Table**. For amplification, the biotin/ tyramine method was used: biotinylated secondary antibody was used as described above before applying ABC Elite Kit peroxidase (#PK-6100, Vector Laboratories) and working buffer containing 0.015% H_2_O_2_ and 7.5 nmol/l biotinylated tyramine. Streptavidin-coupled dyes (#S11223, #S21381, Invitrogen) were used at 1:500 in working buffer for detection. BrdU was detected as described [59]. Micrographs were taken with LSM510 Meta or LSM900 (Zeiss). Images were processed with Adobe Photoshop 2020.

### Fluorescence-activated cell sorting (FACS) and qPCR

For FACS, we pooled the dorsal telencephalon of two E12.5 *Tbr2* GFP tdT reporter mice per sample and dissociated the tissue using Miltenyi Kit #130-094-802 in combination with the gentleMACS Octo dissociator according to the manufacturer’s protocol. The dissociated cells were centrifuged at 300 x *g* for 7 minutes at 4°C, the supernatant was discarded before cells were resuspended in PBS and stained with Zombie Violet dye (Biolegend, 1:200) for dead cell exclusion. After washing with FACS buffer, the cells were passed through a 40 μm strainer before sorting with BD FACSAria Fusion. Single live cells were gated on the basis of dead cell exclusion (Zombie Violet) and doublet exclusion using forward scatter (FSC-W against FSC-A). Four different populations were sorted based on tdT against GFP and lysed in RLT buffer (Qiagen) containing ß-Mercaptoethanol. Samples were snap-frozen in liquid nitrogen and stored at -80°C. Total RNA was isolated from sorted cells using RNeasy-plus Micro Kit (Qiagen #74034) and from embryonic telencephalon/ P30 cerebral cortex using peqGOLD TriFast (VWR #30–2010). Reverse transcription was done using Superscript IV (Invitrogen #18090050), peqGOLD dNTP-Mix (VWR #732–3180), and Oligo-dT (Thermo Fisher #18418020) using 5 μg of total RNA for all tissues and 100–250 ng of total RNA for sorted cells. qPCR for *Donson*, *Tbr2*, *Pax6*, and *Gapdh* was run on a qTower (Analytik Jena) using TaqMan Advanced Mastermix (ThermoFisher Scientific #4444556) and TaqMan Gene Expression Assays (Assay-IDs: Mm00659062_m1, Mm00443072_m1, Mm01351985_m1 and Mm99999915_g1). *Donson*, *Tbr2* and *Pax6* expression in sorted cells was normalized to *Gapdh*. Expression levels were expressed as percentage of the overall mean of the transcript before heat map generation using Prism 9 software.

### Image analysis and statistics

For quantitative analysis, confocal images were captured at 40x with the pinhole set to 1 Airy unit. The tile scan option was used when appropriate. Images were imported into Image J [60] or ZEN (Zeiss). In all cell counting experiments, counting was performed in a constant region of interest. The only exceptions are shown in **Figs 2J** and **5I**, where all CC3^+^ cells ventral to the corticostriatal sulcus (**Fig 2J**) and all *Lhx6*^+^ cells in the lGE (**Fig 5I**) were counted irrespective of the area size. The corticostriatal sulcus was used to distinguish between dorsomedial and ventrolateral pallium. Pax6^+^ cells shown in **Fig 2K** were counted in a constant region of interest placed in the VZ immediately ventral to the corticostrital sulcus. Quantification of the Tbr1^+^ area shown in **Fig 2L** was performed ventral to the corticostriatal sulcus after setting a uniform threshold. The tdT^+^ percentage shown in **Fig 4G** was determined for Ki67^+^, Pax6^+^, and Tbr2^+^ cells by evaluating at least 100 cells per mouse. Cell densities were calculated by dividing the number of counted cells by the size of the evaluated area. Statistical tests were calculated using GraphPad Prism 9 software. Measurements and statistics are summarized in **S1–S4 Tables**.

## Supporting information

S1 Fig *Donson* expression in the embryonic telencephalon. (A) *In situ* hybridizations for *Donson* using a digoxigenin-labeled probe on coronal E11.5 brain sections. *Donson* transcripts are detected in the proliferation zones of the telencephalon and thalamus. (B) The scatter plot demonstrates gating to define a GFP^-^ tdT^-^ population (1) and a GFP^+^ tdT^high^ population (3) in dissociated dorsal telencephalon of E12.5 *Tbr2* reporter mice. Histograms show GFP and tdT signals in populations (1) and (3) as % of maximum. Abbreviations: Ctx, cerebral cortex; DP and MP, dorsal and medial pallium; MaZ; mantle zone of ventral telencephalon; mGE and lGE, medial and lateral ganglionic eminence; mPO, medial preoptic area; Th, thalamus. Scale bar: 350 μm (a.).
(TIF)

S2 Fig *Donson* deletion in the *Emx1* lineage reduces the size of the early dorsomedial pallium. (A,B) Images show Nissl-stained serial coronal sections of the telencephalon for *Emx1*-cKO mice (cKO) and control littermates (ctrl) at E11.5 and E12.5 from rostral to caudal. The dorsomedial telencephalon of E12.5 cKO mice is of regular size at E11.5 (a.), but exhibits a prominent size reduction at mid and caudal sectional planes at E12.5. Insets in (b.) demonstrate cells in the dorsal pallium at high magnification, note presumptive apoptotic bodies in the cKO (arrowheads). The mGE, lGE, cGE, and thalamus appear normal in cKO mice. (C) High magnifications show Nissl-stained E11.5 dorsal telencephalon and overlying the cranium. (D) CC3 immunofluorescence in E11.5 dorsal pallium. Abbreviations: DP, dorsal pallium; MP, medial pallium; mGE, lGE, and cGE, medial, lateral, and caudal ganglionic eminence; Th, thalamus; VZ, ventricular zone. Scale bars: 200 μm (a. and b.); 160 μm (c.), 25 μm (d.).
(TIF)

S3 Fig *Emx1*-cKO mice lack the neocortex. (A-J) Images show coronal sections from *Emx1*-cKO (cKO) and control (ctrl) mice at the indicated embryonic stages. (A) Immunostaining for Tbr1 at rostral (a.), mid (b.), and caudal (c.) sectional planes. (D,E) Anti-Ki67 immunostaining reveals the proliferation zones at rostral (d.) and mid (e.) planes. (F) Anti-Tbr1 immunostaining at a rostral sectional plane. (G) Anti-tdT immunostaining in cKO and ctrl mice containing a *Rosa26*^CAG-LSL-tdT^ allele. Note that the olfactory tract (ot) and the tdT^+^ pallium are shifted dorsally in the cKO. (H) H&E staining of coronal head sections at rostral and caudal sectional planes demonstrate that E16.5 *Emx1*-cKO mice lack the dorsomedial pallium. The CP is absent in the mutant lateral cortex. Arrows point to the lateral neocortex in the ctrl and the corresponding region in the mutant. Note expansion of the ventral subarachnoid space (SAS) in the mutant at the caudal plane. (I,J) Anti-BrdU immunostaining in E16.5 mice receiving a BrdU pulse on E11.5. Images show BrdU^+^ cells in the lateral neocortex (i.) and the piriform cortex/ subpallium boundary zone (j.) of a ctrl and in the corresponding regions of a cKO. (K) Scatter plots show the density of BrdU^+^ cells in the lateral and ventral cortical areas shown in (i.) and (j.). Values are expressed as percentage of the ctrl mean. Circles and triangles represent individual mice, horizontal lines represent the median. Measurements and statistics are summarized in **S1G Table**. Abbreviations: CP, cortical plate; DP, LP, MP, and VP, dorsal, lateral, medial, and ventral pallium; mGE, lGE, and cGE, medial, lateral, and caudal ganglionic eminence; MZ, marginal zone; Pir, piriform cortex; SAS, subarachnoid space; SbP, subpallium; Th, thalamus; ac, anterior commissure; ot, olfactory tract. Scale bars: 200 μm (a. and d.), 400 μm (f. and g.), 64 μm (i.).
(TIF)

S4 Fig cIN accumulate in the ventrolateral cortex of *Emx1*-cKO mice. (A-F) Images and graphs represent E16.5 *Emx1*-cKO (cKO) and control mice. (A,B) Darkfield micrographs show *Lhx6* (a.) and *Reelin* (b.) in emulsion-dipped E16.5 coronal head sections after *in situ* hybridization with ^35^S-labeled probes. Brains are shown at a rostral and at a caudal sectional plane. (C,D) Graphs show quantifications of the hybridization signals of *Lhx6* and *Reelin* in the lateral cortex; the quantification area is indicated by arrowheads in (a.) and (b.). Values represent the positive area fraction for 10 bins (bin 1 corresponds to the MZ) and are presented as mean+SEM. Micrographs in (c.) show *Lhx6* in the lateral cortex. (E) Confocal images demonstrate Reelin in the lateral cortex (i.e. slightly ventral to the corticostriatal sulcus). (F) Images demonstrate eGFP^+^ cGE-derived cIN in E16.5 5HT3-eGFP transgenic *Emx1*-cKO and control mice. Photographs show the lateral cortex (slightly ventral to the corticostriatal sulcus) and ventral cortex (dorsal to the rhinal fissure). The scatter plot shows the density of eGFP^+^ cells in the lateral region. Circles and triangles represent individual mice, horizontal lines represent the median. Horizontal lines indicate the median. Measurements and statistics are summarized in **S1H Table**. **Abbreviations:** CP, cortical plate; dTh and vTH, dorsal and ventral thalamus; GE, ganglionic eminence; LP and VP, lateral and ventral pallium; Mn, meninx; MZ, marginal zone; PSB, pallial/ subpallial boundary; SP, subplate; SVZ, subventricular zone; VG, ventral lateral geniculate; ZLI, zona limitans intrathalamica. Scale bars: 200 μm (a.), 100 μm (c.), 50 μm (e.), 75 μm (f.).
(TIF)

S5 Fig Normal corticogenesis in *Tbr2*-cKO mice. (A) Epifluorescence images demonstrate immunostaining for tdT in P0 *Tbr2*-cKO (cKO) and control (ctrl) mice carrying a *Rosa26*^CAG-LSL-tdT^ allele. Note normal morphology of the cortex and hippocampus in the cKO. (B) Cortical thickness was measured in the M1 region of cKO and ctrl mice at the indicated developmental stages. Circles and triangles represent individual mice. Measurements are provided in **S4 Table**. Scale bar: 200 μm (a.).
(TIF)

S1 Table Data and statistics shown in Figs 1–6, S3 and S4. The table shows measurements and statistics presented in the graphs of **Figs 1–**6**, S3 and S4**.
(XLSX)

S2 Table Number of tdT+ cells in the cortex of *Nkx2.1*-cKO mice at E14.5. The table shows the number of tdT^+^ cells in the cortex of *Nkx2*.*1*-cKO (cKO) and control mice at E14.5. Data are presented in percent of the control mean. The quantification area is indicated by an arrowhead in **Fig 5A**.
(XLSX)

S3 Table Number of Olig2+ cells in the mantle zone of the lGE of E14.5 *Nkx2.1*-cKO mice. The table shows the number of Olig2^+^ cells in the mantle zone of the lGE for *Nkx2*.*1*-cKO (cKO) and control mice at E14.5. Data are presented in percent of the control mean.
(XLSX)

S4 Table Cortical thickness in *Tbr2*-cKO mice. The table shows the cortical thickness for the M1 region of *Tbr2*-cKO (cKO) and control mice. Data are presented in percent of the control mean.
(XLSX)

S5 Table List of Antibodies. The table shows the primary antibodies used in this study, their source and product number, the species they were raised in, and the dilution they were used at. Some antibodies were used at higher dilution when used with biotin tyramine enhancement (enhanced).
(XLSX)
