# Supplementary figures and images for: The microcephaly gene Donson is essential for progenitors of cortical glutamatergic and GABAergic neurons
Source: PLoS Genet. 2021 Mar 19;17(3):e1009441. doi: 10.1371/journal.pgen.1009441 (PMC8011756; doi:10.1371/journal.pgen.1009441)

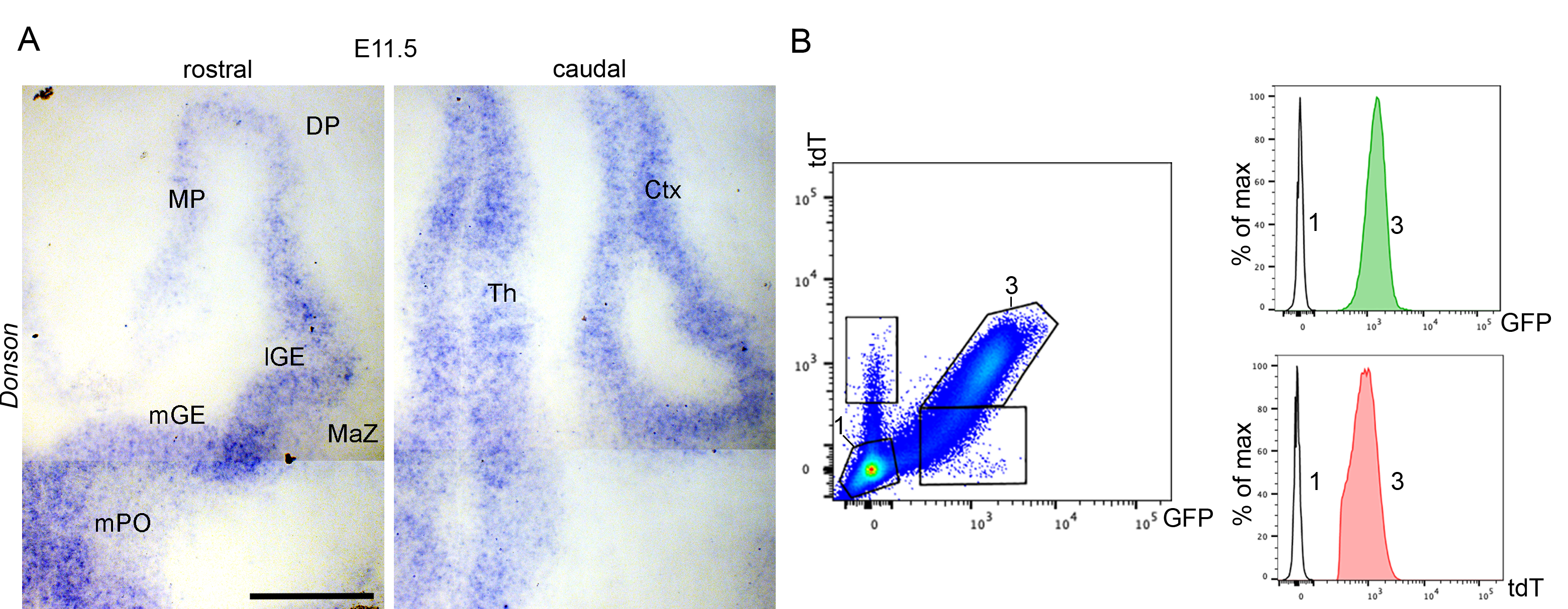

Supplement: S1 Fig — (A) In situ hybridizations for Donson using a digoxigenin-labeled probe on coronal E11.5 brain sections. Donson transcripts are detected in the proliferation zones of the telencephalon and thalamus. (B) The scatter plot demonstrates gating to define a GFP- tdT- population (1) and a GFP+ tdThigh population (3) in dissociated dorsal telencephalon of E12.5 Tbr2 reporter mice. Histograms show GFP and tdT signals in populations (1) and (3) as % of maximum. Abbreviations: Ctx, cerebral cortex; DP and MP, dorsal and medial pallium; MaZ; mantle zone of ventral telencephalon; mGE and lGE, medial and lateral ganglionic eminence; mPO, medial preoptic area; Th, thalamus. Scale bar: 350 μm (a.). (TIF) [file pgen.1009441.s001.tif]

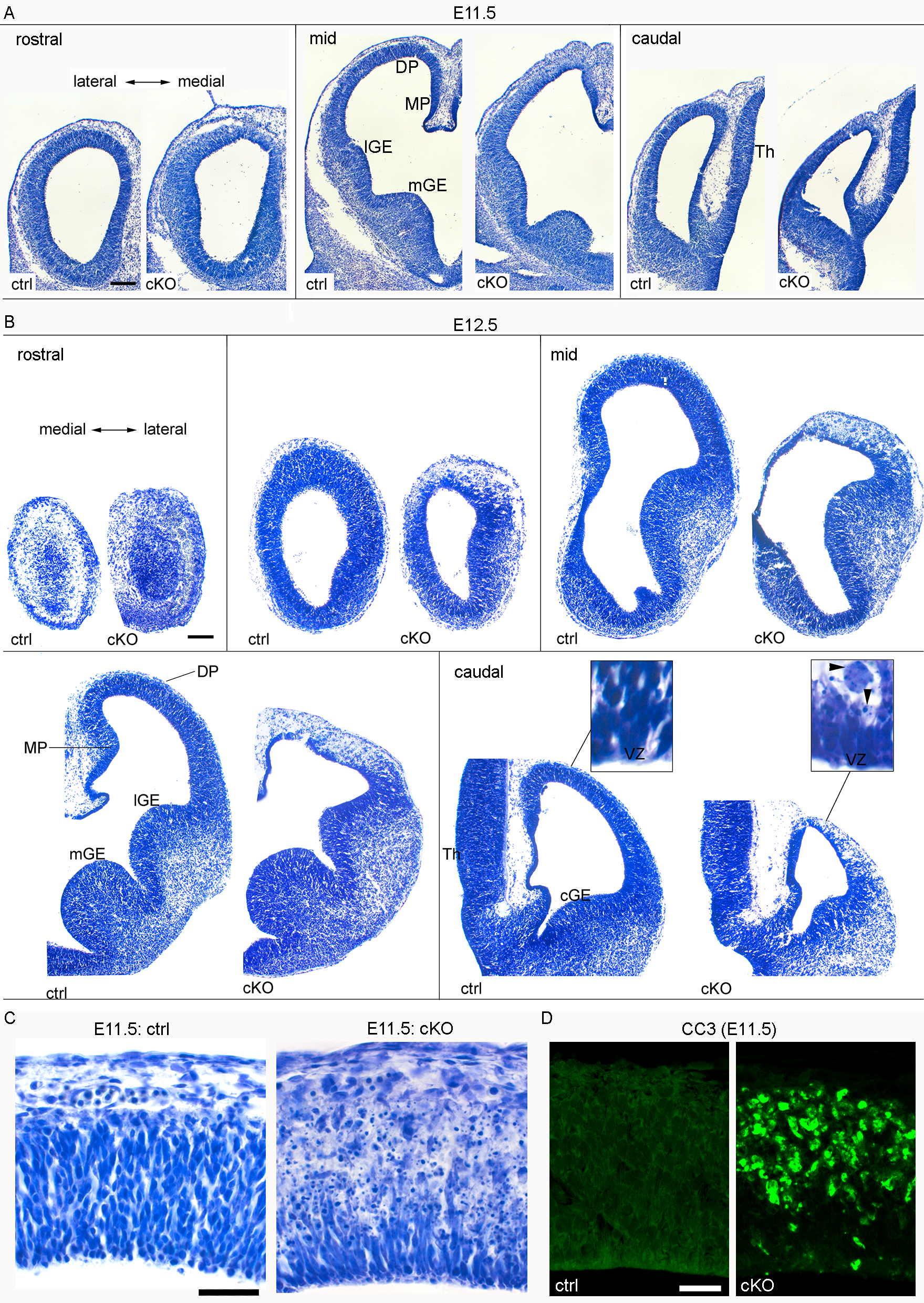

Supplement: S2 Fig — (A,B) Images show Nissl-stained serial coronal sections of the telencephalon for Emx1-cKO mice (cKO) and control littermates (ctrl) at E11.5 and E12.5 from rostral to caudal. The dorsomedial telencephalon of E12.5 cKO mice is of regular size at E11.5 (a.), but exhibits a prominent size reduction at mid and caudal sectional planes at E12.5. Insets in (b.) demonstrate cells in the dorsal pallium at high magnification, note presumptive apoptotic bodies in the cKO (arrowheads). The mGE, lGE, cGE, and thalamus appear normal in cKO mice. (C) High magnifications show Nissl-stained E11.5 dorsal telencephalon and overlying the cranium. (D) CC3 immunofluorescence in E11.5 dorsal pallium. Abbreviations: DP, dorsal pallium; MP, medial pallium; mGE, lGE, and cGE, medial, lateral, and caudal ganglionic eminence; Th, thalamus; VZ, ventricular zone. Scale bars: 200 μm (a. and b.); 160 μm (c.), 25 μm (d.). (TIF) [file pgen.1009441.s002.tif]

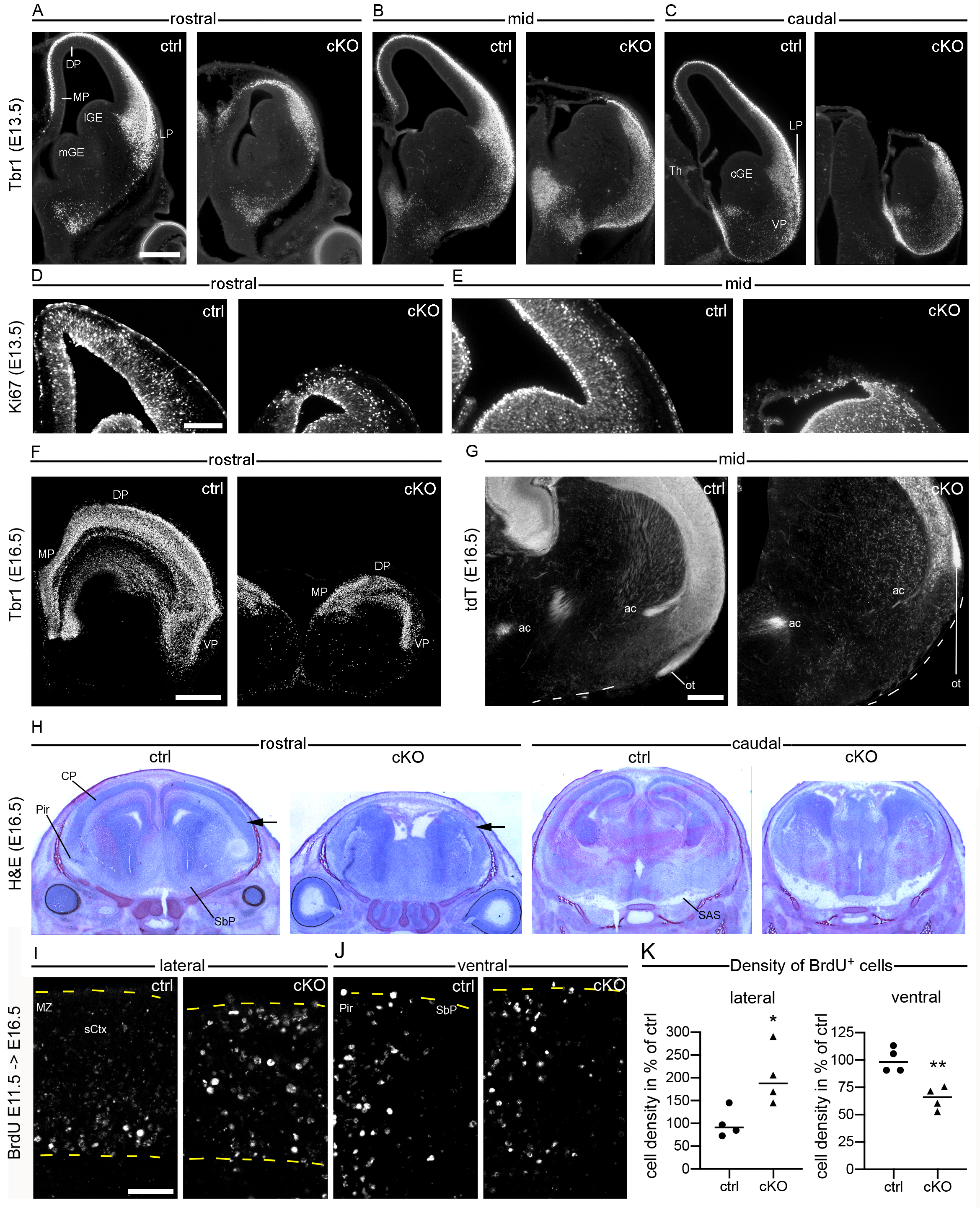

Supplement: S3 Fig — (A-J) Images show coronal sections from Emx1-cKO (cKO) and control (ctrl) mice at the indicated embryonic stages. (A) Immunostaining for Tbr1 at rostral (a.), mid (b.), and caudal (c.) sectional planes. (D,E) Anti-Ki67 immunostaining reveals the proliferation zones at rostral (d.) and mid (e.) planes. (F) Anti-Tbr1 immunostaining at a rostral sectional plane. (G) Anti-tdT immunostaining in cKO and ctrl mice containing a Rosa26CAG-LSL-tdT allele. Note that the olfactory tract (ot) and the tdT+ pallium are shifted dorsally in the cKO. (H) H&E staining of coronal head sections at rostral and caudal sectional planes demonstrate that E16.5 Emx1-cKO mice lack the dorsomedial pallium. The CP is absent in the mutant lateral cortex. Arrows point to the lateral neocortex in the ctrl and the corresponding region in the mutant. Note expansion of the ventral subarachnoid space (SAS) in the mutant at the caudal plane. (I,J) Anti-BrdU immunostaining in E16.5 mice receiving a BrdU pulse on E11.5. Images show BrdU+ cells in the lateral neocortex (i.) and the piriform cortex/ subpallium boundary zone (j.) of a ctrl and in the corresponding regions of a cKO. (K) Scatter plots show the density of BrdU+ cells in the lateral and ventral cortical areas shown in (i.) and (j.). Values are expressed as percentage of the ctrl mean. Circles and triangles represent individual mice, horizontal lines represent the median. Measurements and statistics are summarized in S1G Table. Abbreviations: CP, cortical plate; DP, LP, MP, and VP, dorsal, lateral, medial, and ventral pallium; mGE, lGE, and cGE, medial, lateral, and caudal ganglionic eminence; MZ, marginal zone; Pir, piriform cortex; SAS, subarachnoid space; SbP, subpallium; Th, thalamus; ac, anterior commissure; ot, olfactory tract. Scale bars: 200 μm (a. and d.), 400 μm (f. and g.), 64 μm (i.). (TIF) [file pgen.1009441.s003.tif]

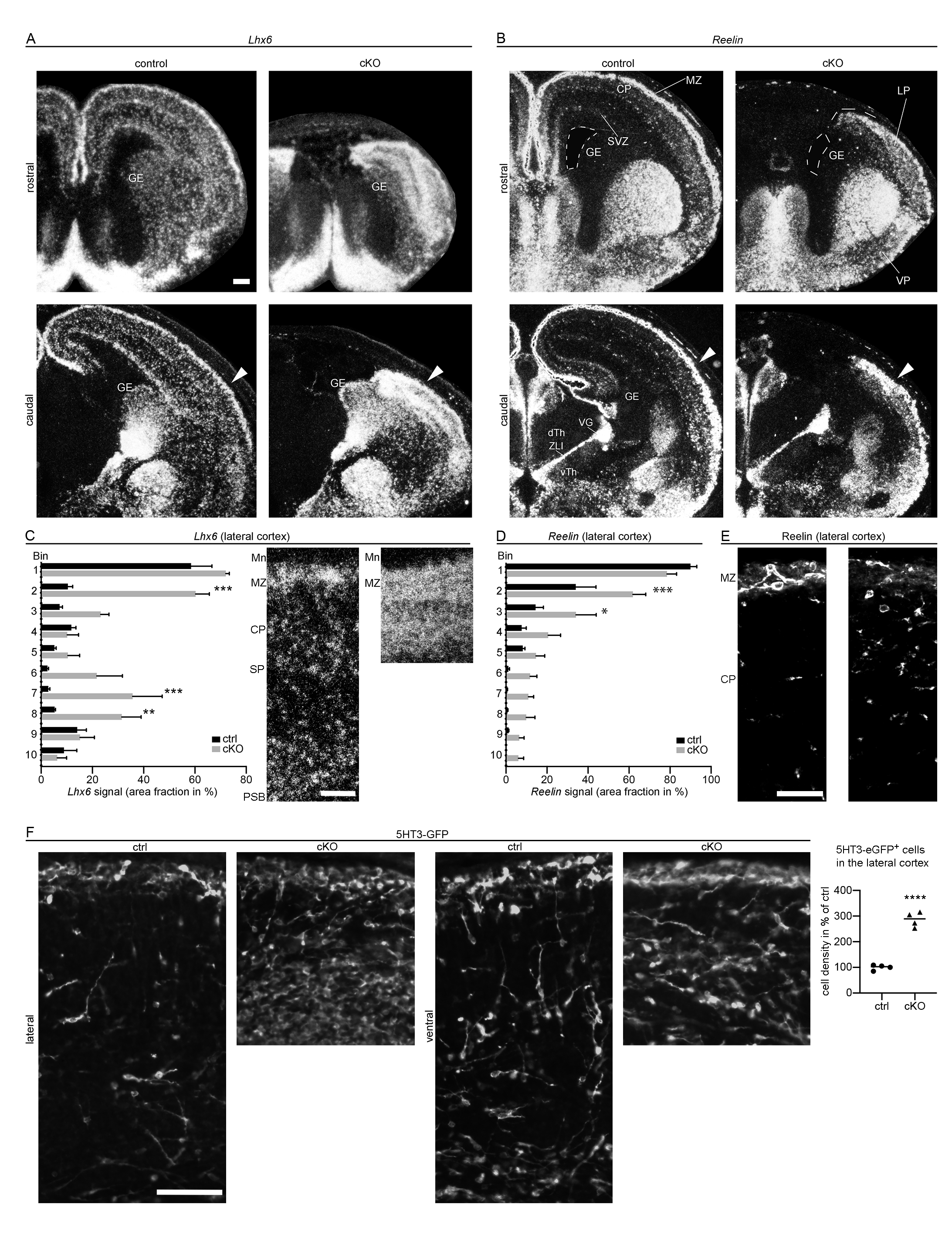

Supplement: S4 Fig — (A-F) Images and graphs represent E16.5 Emx1-cKO (cKO) and control mice. (A,B) Darkfield micrographs show Lhx6 (a.) and Reelin (b.) in emulsion-dipped E16.5 coronal head sections after in situ hybridization with 35S-labeled probes. Brains are shown at a rostral and at a caudal sectional plane. (C,D) Graphs show quantifications of the hybridization signals of Lhx6 and Reelin in the lateral cortex; the quantification area is indicated by arrowheads in (a.) and (b.). Values represent the positive area fraction for 10 bins (bin 1 corresponds to the MZ) and are presented as mean+SEM. Micrographs in (c.) show Lhx6 in the lateral cortex. (E) Confocal images demonstrate Reelin in the lateral cortex (i.e. slightly ventral to the corticostriatal sulcus). (F) Images demonstrate eGFP+ cGE-derived cIN in E16.5 5HT3-eGFP transgenic Emx1-cKO and control mice. Photographs show the lateral cortex (slightly ventral to the corticostriatal sulcus) and ventral cortex (dorsal to the rhinal fissure). The scatter plot shows the density of eGFP+ cells in the lateral region. Circles and triangles represent individual mice, horizontal lines represent the median. Horizontal lines indicate the median. Measurements and statistics are summarized in S1H Table. Abbreviations: CP, cortical plate; dTh and vTH, dorsal and ventral thalamus; GE, ganglionic eminence; LP and VP, lateral and ventral pallium; Mn, meninx; MZ, marginal zone; PSB, pallial/ subpallial boundary; SP, subplate; SVZ, subventricular zone; VG, ventral lateral geniculate; ZLI, zona limitans intrathalamica. Scale bars: 200 μm (a.), 100 μm (c.), 50 μm (e.), 75 μm (f.). (TIF) [file pgen.1009441.s004.tif]

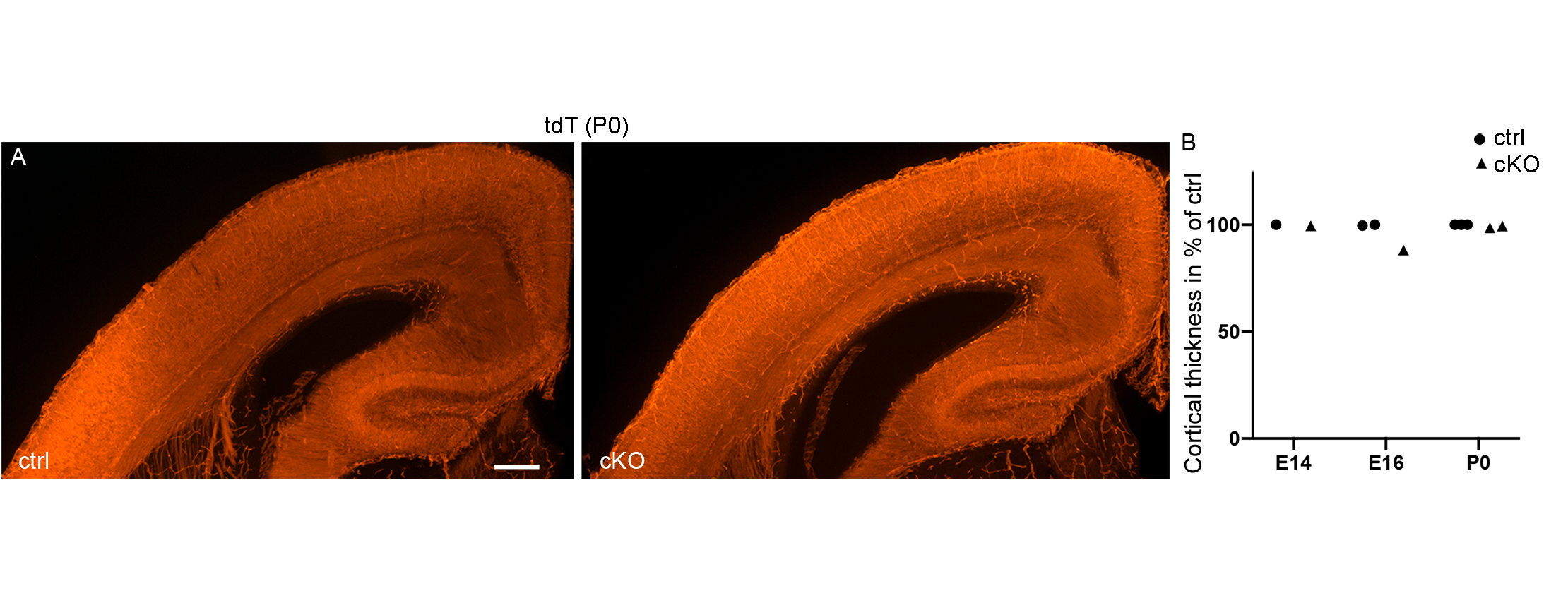

Supplement: S5 Fig — (A) Epifluorescence images demonstrate immunostaining for tdT in P0 Tbr2-cKO (cKO) and control (ctrl) mice carrying a Rosa26CAG-LSL-tdT allele. Note normal morphology of the cortex and hippocampus in the cKO. (B) Cortical thickness was measured in the M1 region of cKO and ctrl mice at the indicated developmental stages. Circles and triangles represent individual mice. Measurements are provided in S4 Table. Scale bar: 200 μm (a.). (TIF) [file pgen.1009441.s005.tif]
